# Implementation of negative pressure for acute pediatric burns (INPREP): A stepped-wedge cluster randomized controlled trial protocol

**DOI:** 10.1371/journal.pone.0315278

**Published:** 2024-12-10

**Authors:** Maleea D. Holbert, Fiona Wood, Andrew J. A. Holland, Warwick Teague, Roy M. Kimble, Dianne Crellin, Cody C. Frear, Kristen Storey, Natalie Phillips, Yvonne Singer, Tanesha A. Dimanopoulos, Lisa Martin, Leila Cuttle, Dimitrios Vagenas, Steven M. McPhail, Pauline Calleja, Jed Duff, Alexandra De Young, Bronwyn R. Griffin

**Affiliations:** 1 School of Nursing and Midwifery, Griffith University, Nathan Campus, South Brisbane, QLD, Australia; 2 Children’s Health Queensland Hospital and Health Service, South Brisbane, QLD, Australia; 3 Perth Children’s Hospital, Nedlands, WA, Australia; 4 Burn Injury Research Unit, University of Western Australia, Crawley, WA, Australia; 5 The Burns Unit, The Children’s Hospital at Westmead, Westmead, NSW, Australia; 6 Faculty of Medicine and Health, Sydney Medical School, The University of Sydney, Sydney, NSW, Australia; 7 Murdoch Children’s Research Institute, Surgical Research, Parkville, VIC, Australia; 8 Burns Service, The Royal Children’s Hospital Melbourne, Parkville, VIC, Australia; 9 Department of Paediatrics, The University of Melbourne, Parkville, VIC, Australia; 10 School of Public Health & Preventive Medicine, Monash University, Melbourne, VIC, Australia; 11 Faculty of Medicine, University of Queensland, Herston, Brisbane, QLD, Australia; 12 Department of Nursing, The University of Melbourne, Parkville, Victoria, Australia; 13 Child Health Research Centre, University of Queensland, South Brisbane, QLD, Australia; 14 Faculty of Health, School of Biomedical Sciences, Queensland University of Technology, Brisbane, Australia; 15 Faculty of Health, Research Methods Group, School of Public Health and Social Work, Queensland University of Technology, Brisbane, Australia; 16 Australian Centre for Health Services Innovation and Centre for Healthcare Transformation, School of Public Health and Social Work, Queensland University of Technology, Brisbane, Kelvin Grove, Australia; 17 College of Healthcare Sciences, James Cook University, Cairns, Australia; 18 Faculty of Health, Centre for Healthcare Transformation, Queensland University of Technology, Brisbane, Australia; University of California Davis, UNITED STATES OF AMERICA

## Abstract

**Background:**

Acute application of adjunctive negative pressure wound therapy (NPWT) significantly improves time to re-epithelialization in pediatric burn patients. This adjunctive treatment has not yet been broadly or routinely adopted as a standard primary burns dressing strategy. The Implementation of Negative PRessurE for acute Pediatric burns (INPREP) trial will implement and evaluate the impact of adjunctive NPWT in parallel with co-designed implementation strategies and resources across four major pediatric hospitals.

**Methods:**

We will conduct a multi-center, prospective, stepped-wedge cluster randomized controlled trial to implement adjunctive NPWT for acute pediatric burns. Participants will include pediatric burn patients presenting to one of four Australian tertiary pediatric hospitals for burn treatment. The intervention is adjunctive NPWT in parallel with co-designed and tailored implementation strategies and a suite of NPWT implementation resources, which form the INPREP toolkit. Using a hybrid type III design, this trial aims to evaluate the effectiveness of NPWT implementation in parallel with the INPREP toolkit using (i) implementation outcomes (e.g., adoption, appropriateness, acceptability, feasibility, and sustainability) and (ii) clinical outcomes (e.g., days to re-epithelialization, scar management requirements, skin grafting requirements). The primary outcome of this trial is treatment adoption–the proportion of eligible patients who receive NPWT.

**Discussion:**

This manuscript outlines a protocol for a hybrid type III stepped-wedge cluster randomized controlled trial of adjunctive NPWT implementation in acute pediatric burn care. We anticipate that NPWT implementation in parallel with the INPREP toolkit will be generalizable to emergency departments and burn services across Australia, and evidence generated will inform pediatric burn care internationally.

**Trial registration:**

Australian and New Zealand Clinical Trials Registry: ACTRN12622000166774. Registered 1 February 2022.

## Introduction

Pediatric burns pose a major public health concern worldwide, with burn injuries ranked as the fifth most common cause of non-fatal injuries in children [1,2]. Each year, over 700 children across Australia sustain acute burn injuries that require inpatient admission and definitive wound management from a dedicated burn service [3,4]. Research indicates that between 16%– 35% of pediatric burn patients develop hypertrophic scarring [5–9], which is associated with poor long-term outcomes such as significant functional, cosmetic, and psychosocial impairment [10–12]. Time to re-epithelialization is a significant predictor of hypertrophic scarring in patients following a burn [9]. Therefore, a critical goal of burn care is to minimize healing time and achieve rapid re-epithelialization of the burn wound. Despite significant advances in the treatment of burn injuries, including evidence-based first aid and silver-impregnated dressings [13–17], serious risks of poor long-term outcomes for children remain.

Negative pressure wound therapy (NPWT) is a wound dressing system that provides sub-atmospheric pressure within a closed dressing; a battery-powered pump or computerized device produces a controlled suction to the wound bed via a connective port. This treatment, also referred to as vacuum-assisted closure, is associated with improved patient outcomes across a spectrum of complex chronic, surgical, and acute wounds [18–26]. NPWT facilitates wound healing through various mechanisms, such as inducing macrodeformation (i.e., wound contraction) and microdeformation (i.e., interactions between tissue and dressing at the microscopic level), promoting angiogenesis in the wound bed, improving microvascular blood flow and tissue perfusion, stimulating growth of granulation tissue, decreasing oedema, controlling wound exudate through improved fluid drainage, and reducing risk of infection via decreasing bacterial loads [27–30].

Empirical evidence from experimental [31,32], randomized controlled trials [33–37], and prospective cohort studies [38–40] support the advantages and clinical benefits of adjunctive NPWT in acute burn care. In comparison to standard silver dressings alone, acute adjunctive NPWT (i.e., applied on top of standard silver dressings within 48 hours post-burn) results in significant improvements in time to re-epithelialization, reductions in the need for scar management, and operating theatre time for pediatric patients with acute burn injuries [34]. Furthermore, acute adjunctive NPWT can offer a cost-effective solution for the treatment of pediatric burns. One recent investigation evaluating the healthcare costs of adjunctive NPWT in small-area pediatric burns found the mean total cost per person for the standard silver dressings group was $1,669 AUD (95% CI 659–3269), compared to $904 AUD (95% CI 671–1235) for the NPWT group [41]. Despite evidence supporting the use of NPWT for acute burn management, evidence-based guidelines for incorporation of NPWT into acute pediatric burn care have not yet been developed or disseminated. We propose to facilitate the early implementation of NPWT into emergency department (ED) and acute burn care practices to provide Australian children early access to evidence-based treatment that may improve clinical outcomes and reduce healthcare costs.

Partnering with four major pediatric Australian hospitals, the first phase of this research was to co-design implementation strategies tailored to local contexts to support earlier and/or greater use of adjunctive NPWT for the treatment of acute pediatric burn injuries and develop a suite of NPWT implementation resources–forming the NPWT implementation toolkit (referred to as the INPREP toolkit) to be used in this trial [42]. This manuscript outlines the protocol for the SW-RCT. We hypothesize that facilitated implementation will improve adoption of early adjunctive NPWT application in acute burn care settings (i.e., ED, operating theatre, burn center, and ward). It is further hypothesized that pediatric burn patients who receive adjunctive NPWT during their acute burn care will show improvements in time to re-epithelialization, decreased scar management requirements, and a reduction in surgical interventions (such as skin grafting) compared to those patients who do not receive acute adjunctive NPWT.

### Aim and objective

The aim of this investigation is to examine the effectiveness of acute adjunctive NPWT implementation in parallel with the INPREP toolkit. Moreover, this trial aims to investigate the effects of acute adjunctive NPWT on clinical patient outcomes (e.g., time to re-epithelialization and skin graft requirements) for pediatric patients presenting to hospitals with acute burn injuries. To achieve this aim, we will complete the following two objectives:

1. Assess implementation outcomes (e.g., adoption, acceptability, appropriateness, feasibility, and sustainability).
2. Assess clinical outcomes (e.g., time to re-epithelialization, scar management requirements, skin grafting requirements, and healthcare costs). Findings from these objectives will also be used to inform a trial-based economic evaluation.
3. Examine markers of stress and healing in biospecimens (e.g., levels of inflammatory mediators and stress following a burn injury).

### Primary hypothesis

*We hypothesize that* acute adjunctive NPWT implementation in parallel with the INPREP toolkit *(i*.*e*., *co-designed implementation strategies and tailored resources) will be considered acceptable*, *appropriate*, *and feasible to clinicians*, *patients*, *and carers*, *resulting in application of adjunctive NPWT for the treatment of acute pediatric burn injuries*.

### Secondary hypothesis

Pediatric patients with burns treated in the implementation phase will heal significantly faster, resulting in reduced surgical requirements, scar management referrals, and overall healthcare costs per patient compared to those patients treated in the control phase.

## Methods

### Trial design

Acute adjunctive NPWT, in parallel with the co-designed INPREP toolkit, will be implemented and integrated across four major pediatric Australian hospitals. The effectiveness of this implementation will be evaluated using a hybrid type III effectiveness-implementation design. Within hybrid study designs, research questions assessing both the clinical effectiveness of an intervention as well as the effectiveness of the implementation of the intervention are examined [43,44]. Type III design refers to studies in which the principal focus is the assessment and impact of implementation strategies on implementation outcomes (i.e., acceptability, appropriateness, and feasibility) and clinical effectiveness data is collected concurrently [43]. Hybrid type III designs are utilized when evidence for the intervention has been established [32,34,37,41], and the focus of the evaluation is on implementation outcomes [43]. Implementation strategies and clinical patient outcomes will be evaluated via a multi-center, prospective, pragmatic cluster SW-RCT at four pediatric tertiary hospitals (each hospital will constitute one cluster) across four Australian states. The SW-RCT suits the implementation of evidence-based innovations where it is impossible or impractical to enroll half of participating sites to the intervention arm due to pragmatic and logistic issues, allowing all sites to eventually receive the intervention (i.e., adjunctive NPWT implementation in parallel with the INPREP toolkit) during the trial [45,46]. We aim to evaluate the impact of adjunctive NPWT in parallel with the INPREP toolkit on implementation outcomes and patient outcomes over three periods (control, implementation, and sustainability).

### Setting

This SW-RCT will be conducted within four major Australian pediatric hospitals located in New South Wales, Queensland, Victoria, and Western Australia. Each pediatric hospital is the nominated statewide burn referral center for their respective state. Pediatric burn patients can be recruited for this trial within the ED, burn center, ward, and operating theatre. Human Research Ethics Committee (HREC) approval has been obtained for this investigation (HREC/21/QCHQ/81002) from Children’s Health Queensland Hospital and Health Service HREC on 21 December 2021.

### Intervention

The intervention will be the implementation of acute adjunctive NPWT in parallel with the INPREP toolkit. NPWT devices used across participating hospitals include the PICO™ 7 Single Use Negative Pressure Wound Therapy System (Smith & Nephew Medical Ltd.), RENASYS TOUCH™ (Smith & Nephew Medical Ltd), AVELLE™ Negative Pressure Wound Therapy System (ConvaTec Inc.) and 3M™ ActiV.A.C.™ Therapy System (KCI USA, Inc.). Regarding the minimum treatment duration, the NPWT dressing is to remain in situ for a minimum of 3 days and maximum of 7 days, unless the treating team elects to cease earlier for clinical reasons. If ceasing NPWT prior to the minimum timeframe, the reasons for protocol deviation will be documented. Acute adjunctive NPWT refers to NPWT application within the first 48-hours following the initial burn. A fundamental goal of adjunctive NPWT use within the acute burn phase is to prevent deepening of the burn wound and conversion to deep-dermal and full-thickness injuries, as vascular impairment can persist for up to 48-hours post-burn [47]. Due to the nature of the intervention (i.e., use of a specific medical device), participants and healthcare professionals administering the intervention cannot be blinded. Researchers assessing outcomes in this trial will be masked, as will the statistical team.

### Conceptual frameworks and development of the INPREP toolkit

We drew on a determinant framework, the Consolidated Framework for Implementation Research (CFIR), to guide the development of implementation strategies [48]. This SW-RCT was preceded by a sequential mixed methods phase, referred to as phase one [42]. In phase one, the CFIR was used to inform the planning and development of the INPREP toolkit, encompassing tailored implementation strategies and resources (see Table 1). A purpose-built online questionnaire was developed and disseminated to healthcare professionals involved in the acute management of pediatric burn patients across the four participating sites–to determine perceived barriers to NPWT use and implementation into acute burn care. Identified CFIR barriers were matched to relevant implementation strategies using the CFIR-ERIC matching tool [49]. Following this, online and in-person semi-structured interviews were conducted with senior clinicians at participating sites to generate tailored implementation strategies specific to local hospital and health service contexts. As mentioned, these tailored and co-designed implementation strategies formed the INPREP toolkit. Full methods and results for phase one have been published [42].

**Table 1:** INPREP toolkit components.

Component	Focus	Format	Target
Education	I. NPWT education and troubleshooting guide	Paper and electronic	Parents-caregivers
	II. NPWT clinical education document–outlining commencement and management of NPWT	Paper and electronic	Clinicians
	III. NPWT instructional video–demonstrating adjunctive NPWT application using two different NPWT devices to different anatomical regions	Online	Clinicians
	IV. NPWT practical in-service education and training sessions	In-person	Clinicians
Communication	V. Regular research meetings	In-person and online	Clinicians
	VI. Identification of clinical implementation champions		
Guidelines	VII. NPWT Decision Pathway	Paper and electronic	Clinicians
	VIII. Development of inclusion and exclusion criteria for adjunct NPWT for acute pediatric burn injuries	Paper and electronic	Clinicians

Master versions of the developed implementation resources are provided in S1–S3 Files.

### Participants

Inclusion and exclusion criteria for the use of adjunctive NPWT in children with acute burn injuries were established using online questionnaire data, refined and tailored during semi-structured interviews with clinicians, and then consolidated in a consensus group meeting with clinical investigators. A barrier to NPWT implementation identified in the first phase of this investigation was a lack of clear inclusion and exclusion criteria for the use of acute adjunctive NPWT in pediatric burn patients. The development of inclusion and exclusion criteria occurred in phase one [42].

#### Inclusion criteria

Participants recruited into the SW-RCT will include pediatric patients (defined as <16 years old) presenting with an acute burn to a participating hospital within 48-hours post-burn and requiring definitive wound treatment from a burn service. All burn mechanisms are eligible for enrolment in the trial, and there is no minimum or maximum total body surface area (TBSA) percentage.

#### Exclusion criteria

Exclusion criteria include eye burns, patients with known allergies to adhesive fixation, superficial (erythema only) burns, and a broad criterion referred to as patient factors and contextual considerations limiting NPWT usage. We have created this broad exclusion criterion to allow treating clinicians to decide if acute adjunctive NPWT use is appropriate and suitable for their patients. Potential examples of patient factors and contextual considerations for exclusion in the trial include children with pre-existing diagnoses (behavioral, cognitive, or developmental difficulties) limiting compliance or use of the NPWT device, circumferential deep-dermal or full-thickness burns excluded at the discretion of treating clinicians to assess capillary refill, and complex social and environmental circumstances (i.e., households without access to electricity to charge the devices, or rural and remote families with limited local support). Some patients with pre-existing diagnoses, deep and full-thickness circumferential burns, and complex social and environmental circumstances might still be suitable for enrolment into the trial, and this pragmatic and flexible approach allows the treating team to determine if participation in the trial is appropriate.

### Study outcomes

#### Primary outcome

The primary outcome of this hybrid type III SW-RCT is adoption of acute adjunctive NPWT implementation as per guideline components included in the INPREP toolkit (see Table 1). Adoption in this context is defined as the proportion (percentage) of patients who received acute adjunctive NPWT (i.e., percentage of eligible pediatric burn patients who received NPWT compared to those who were eligible but did not receive NPWT during the implementation phase) in accordance with the established inclusion and exclusion criteria, and duration of NPWT application defined within the INPREP toolkit.

### Secondary outcomes

#### Secondary implementation outcomes

- *Appropriateness*–clinician and patient-carer perspectives of toolkit and procedure will be assessed using the Intervention Appropriateness Measure [50].
- *Acceptability–*clinician and patient-carer perspectives of toolkit and procedure will be assessed using the Acceptability of Intervention Measure [50].
- *Feasibility–*clinician and patient-carer perspectives of toolkit and procedure will be assessed using the Feasibility of Intervention Measure [50].
- *Fidelity–*defined as NPWT treatment as per the INPREP toolkit. Assessed via the number of documented protocol deviations (i.e., document all incidences where NPWT was not applied within the minimum timeframe post-burn, and cases where NPWT was removed before the minimum treatment timeframe).

#### Secondary patient outcomes

- *Healing outcomes*: i) time (days) to 95% burn wound re-epithelialization, ii) skin grafting requirements, iii) burn wound exudate, iv) number of dressing changes, v) adverse events (e.g., infection, unplanned return for dressings, and vi) pain (assessed via observational and self-report pain measures) [34].
- *Operating theatre requirements*: i) proportion of patients requiring an operation (including dressing changes performed under a general anesthetic), ii) operations required per patient, and iii) operative procedures performed (e.g., skin graft) [34].
- *Hospital requirements 12 months post-injury*: i) proportion of patients needing ≥1 admissions to hospital (yes/no), ii) length of stay (days), iii) number of outpatient appointments, and iv) number of scar clinic appointments.
- *NPWT device malfunctions*: i) alarms (yes/no) and ii) power/charging issues (yes/no)

#### Secondary resource use outcomes (12-month time horizon)

- Implementation costs (e.g., change facilitator, staff time for training, resources, and materials)
- Health care resource use comparing control and intervention groups (e.g., time to apply NPWT, cost of device, dressings used, surgical procedures and interventions, unexpected returns to hospital, and removal and reapplication of NPWT at follow-up dressing change appointments)

### Study procedures

**Fig 1:**
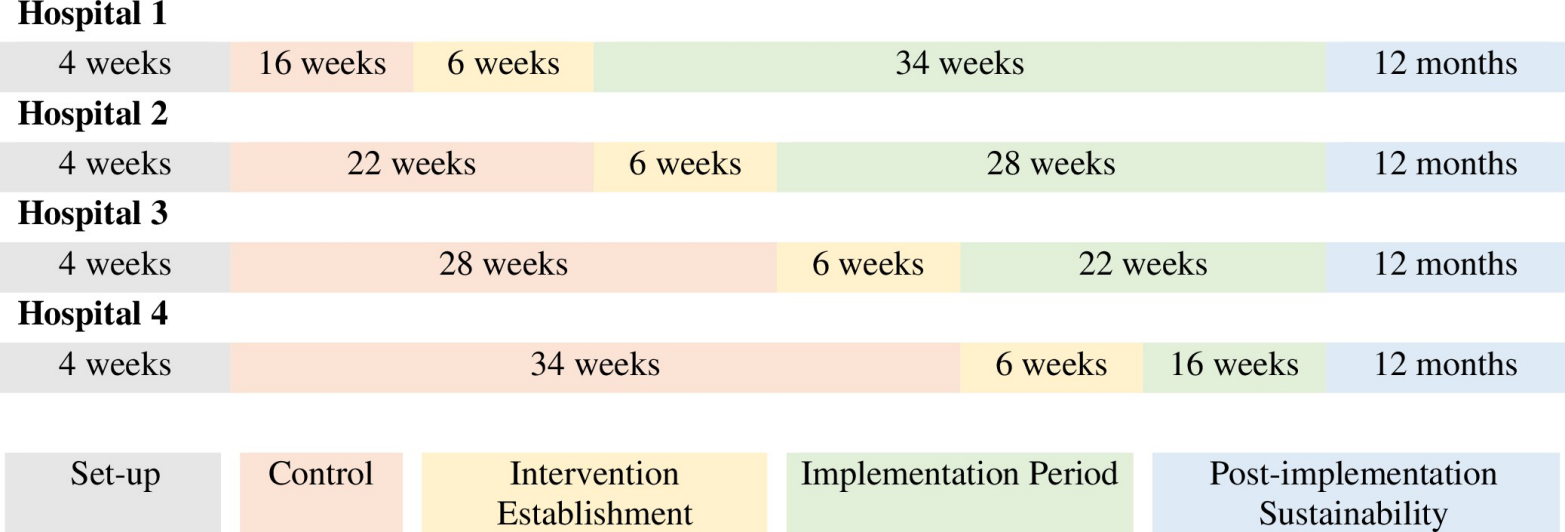
Steps for the multi-center SW-RCT.

Five steps will be sequentially rolled out across the four hospitals over 13 months:

1. **Set up** ensures adequate training and preparation for collection of control and intervention data. Following the initial set-up, all four participating sites (clusters) will start the trial in the control phase with baseline measures taken, and then each site will step up (sites randomly selected via computer-generated, centralized randomization sequence) to implementation every six weeks until saturation of the implementation intervention across all sites. Implementation education will provide evidence-based recommendations–but treating clinicians will determine definitive care (e.g., inpatient versus outpatient care) based on patient needs and healthcare priorities. In both control and implementation steps, other burn care will follow local standards and be determined by treating clinicians [51].
2. **Control step** is usual care (i.e., standard silver dressings).
3. **Intervention establishment** involves research officers (ROs) collaborating with chief investigators and clinicians across the four participating sites to provide targeted NPWT education, practical training in the application and use of adjunctive NPWT for the acute treatment of pediatric burns, promoting acute adjunctive NPWT as part of standard care practices, and delivering additional companion implementation strategies.
4. **Implementation period** Staff initiate acute adjunctive NPWT treatment as per guideline components included in the INPREP toolkit and the implementation of the co-designed implementation strategies (see Table 1).
5. **Sustainability** of adoption continues to be monitored to see if practice was sustained when facilitated implementation stopped. Fig 1 illustrates the SW-RCT design utilized within this trial.

### Ethics approval and consent to participate

Ethics approval has been obtained for this investigation (HREC/21/QCHQ/81002) from Children’s Health Queensland Hospital and Health Service HREC. This research was also approved by the Griffith University Human Research Ethics Committee (GU Ref No: 2022/157).

#### Recruitment of participants

Wherever possible, prospective informed consent will be obtained from parents and caregivers in the ED or burn center following initial patient presentation to a participating hospital. Children will also be included in the consent discussion where age appropriate–a suitably modified version of the information sheet has been developed for older pediatric patients. Parents and caregivers, and the child, where age appropriate, will be approached by a member of the research team and presented with information regarding the trial. Consent to continue (also referred to as deferred consent or research without prior consent) will be used in extenuating circumstances where it is not possible, practical, or appropriate to obtain prospective informed consent. In these circumstances, parents and caregivers will be informed about the research as soon as possible after it commences, and their written consent for continuing involvement will be sought. For patients and families who have provided verbal consent, written consent for continuing involvement will be obtained as close to 24 hours following the hospital presentation as possible–but no later than 72 hours. For children not scheduled to return to burn services within 72 hours following verbal consent, written informed consent will be obtained using a REDCap (Research Electronic Data Capture, Vanderbilt, USA, hosted at Griffith University) link tick box emailed to the parents and carers. It will be made clear that if the parent/caregiver withdraws at that point or anytime thereafter, they will be given the option to have any collected data or samples destroyed. Consent will be to care as usual within the trial phase. Participant recruitment will occur from August 2023 until September 2024.

#### Randomization

All sites will start the trial in the control phase with the baseline measures taken. Following this, as shown in Fig 1, the adjunctive NPWT intervention will be rolled out to each site. For this, a randomization procedure will occur where the sites transition in a random order. The randomization procedure will be performed by the statistician of the study (DV). He will be provided with a masked list of sites (i.e., A, B, C, D) which are linked to the actual site name. He will randomly allocate them to a step in order for them to make the transition from control to treatment. This randomization will be performed in R Statistical Software [52]. At 13 months after implementation has occurred across all sites, adoption sustainability will be measured to inform implementation effectiveness. Due to the nature of the intervention, participants and healthcare professionals administering the intervention cannot be blinded. Therefore, researchers assessing outcomes in this trial and the biostatistician analyzing the data will be masked (i.e., they will not be aware of the treatment each patient receives).

#### Measurement tools

The Acceptability of Intervention Measure (AIM), Intervention Appropriateness Measure (IAM), and Feasibility of Intervention Measure (FIM) are four-item measures of implementation outcomes that are considered fundamental to implementation success. These measures can be administered to determine the extent to which clinicians, as well as parents and caregivers, feel adjunctive NPWT for the acute treatment of pediatric burns is acceptable, appropriate, and feasible. The measures can be used independently or together. The AIM, IAM and FIM have been shown in psychometric studies using content, known groups and responsiveness validation methods to provide reliable and valid assessments of implementation outcomes [53].

#### Data collection

**Fig 2:**
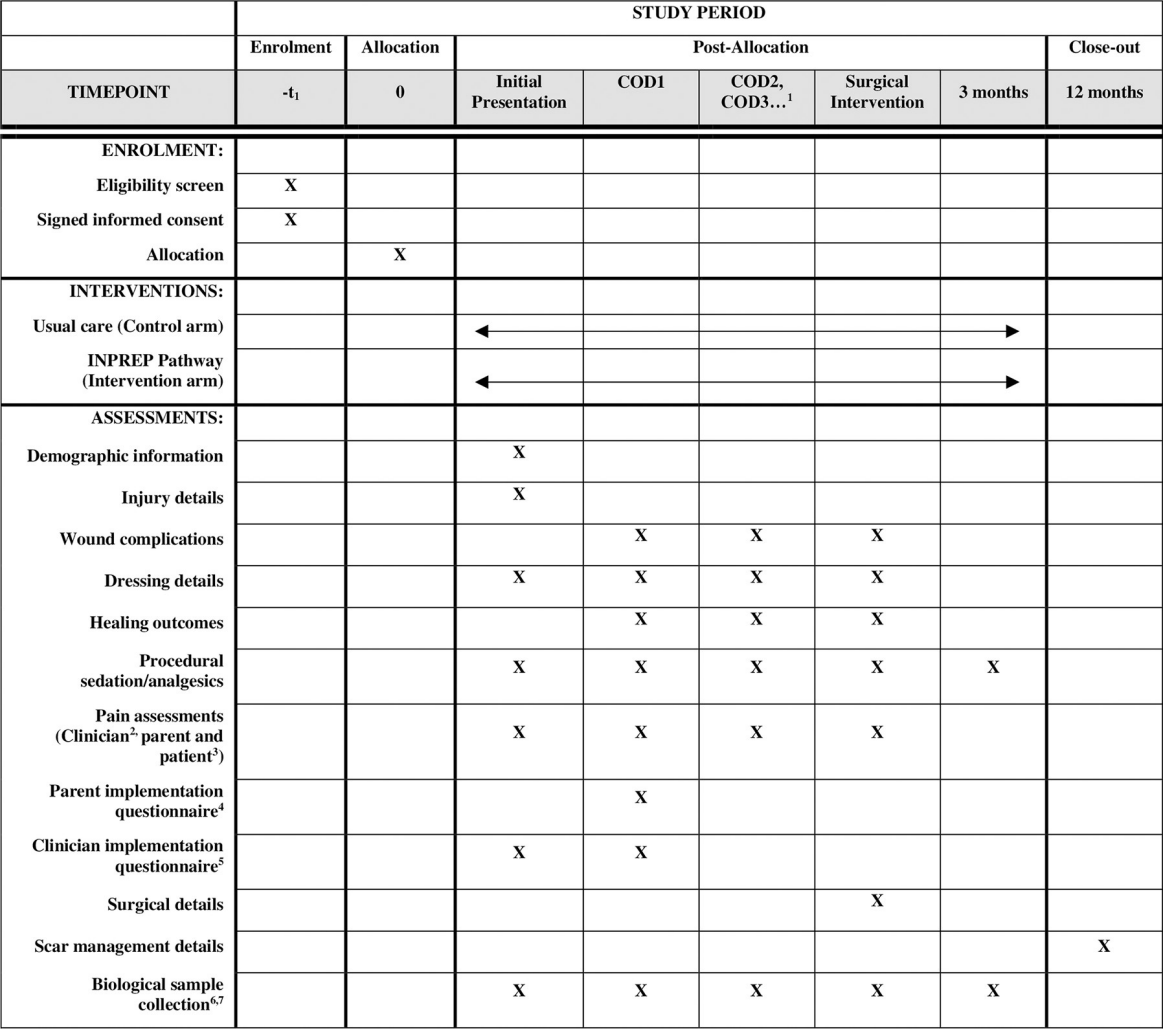
Schedule of enrolment, interventions, and assessments. COD = change of dressing; ^1^ Every change of dressing until patient is 95% re-epithelialised; ^2^ Face, legs, activity, cry, and consolability scale to be collected if patient under 7 years of age, numeric rating scale to be collected if patient aged 7 years or older; ^3^ Patient pain scales only required for patients aged 7 years or older; ^4,5^ Implementation outcomes only required for patients enrolled in the intervention arm; ^6^ Blood sample only if patient is already cannulated or undergoing procedure under a general anesthetic; ^7^ Wound fluid collected at COD1 only if patient in intervention arm and wound fluid able to be collected from NPWT device tubing.

Research officers (ROs) will prospectively collect primary and secondary outcome data using purpose-built REDCap data collection instruments (Research Electronic Data Capture, Vanderbilt, USA, hosted at Griffith University) on password-protected iPhones. REDCap is a secure, web-based software platform designed to support data capture for research studies [54,55]. Each participating site will be provided with an iPhone for data collection. The schedule of assessments and data collection time points for this SW-RCT are presented below in Fig 2. Burn wound photos will be taken with an iPhone following initial presentation to the participating hospital and at each subsequent dressing change until 95% burn wound re-epithelization has occurred. These photos will be used as part of a blinded review for time to healing. The clinician who applies the adjunctive NPWT to participants as part of their acute burn treatment will be asked to assess the acceptability, appropriateness, and feasibility of NPWT use using the AIM, IAM, and FIM [50] via a REDCap form. The same data points will be obtained from the clinician who removes the NPWT. Following the removal of NPWT, parents and caregivers will also be asked to assess the acceptability, appropriateness, and feasibility of NPWT use as part of the INPREP toolkit using the AIM, IAM, and FIM [50].

### Collection of biospecimens

Where collection of biospecimens can be achieved as part of standard care without causing pain or distress, consent to collect a specimen will be sought from participants and their families. Potential biospecimen collection will include blood (while sedated/anesthetized, during IV cannula insertion for fluid therapy, or part of a routine blood draw), wound exudate (during wound debridement or a dressing change), urine (collection in a specimen pot from a continent child, or part of routine urinary catheter use), and/or a hair sample from the patient (4mm diameter of hair strands cut close to the scalp).

- Wound exudate will be collected and stored using the Standard Operating Procedures (SOP) for Wound Fluid (see S4 File). Researchers involved in this trial have previous experience collecting wound exudate from NPWT devices [34] and directly from the wound into collection tubes [56]. If the fluid volume is <0.5mL, filter paper will be placed at the edge of the burn wound to absorb the wound exudate [57,58].
- Blood will be collected, processed, and stored using the SOP for blood processing (see S5 File).
- A hair sample containing approximately 100 strands of hair and a minimum of 3cm in length will be collected from the area at the nape of the neck, cut as close to the scalp as possible using scissors (see S6 File). Hair is a unique biospecimen as it enables a baseline assessment in pediatric burn patients by analyzing the hair which was growing pre-burn. All biospecimens will be stored at -80°C until they are analyzed as a batch.
- Urine samples will be collected, clarified by centrifugation, filtered to remove bacteria or protein aggregates using previously established techniques [59], and stored using the SOP for urine processing (see S7 File).

The purpose of biospecimen analyses is to determine the levels of inflammatory mediators and stress following a burn injury. The exudate from the wound, collected using the NPWT device, will be analyzed using immuno-assays to identify the presence and quantity of inflammatory cytokines. This analysis will provide evidence of the NPWT device’s effectiveness in reducing excessive immune mediators after burn injuries. Additionally, cortisol levels will be measured in hair and urine samples using quantitative Liquid Chromatography Mass Spectrometry (LCMS-MS). The baseline cortisol level will be assessed in hair samples, while urine samples will indicate stress levels following the burn injury. The biospecimen analysis will enhance our understanding of the biological mechanisms underlying the effectiveness of acute adjunctive NPWT use and contribute to the improvement of wound management treatment protocols.

### Healing outcomes measures (secondary outcomes)

#### Time to re-epithelialization

The number of days from the date of the initial burn until 95% wound re-epithelialization occurs will be determined using two methods–clinical judgment from the treating consultant and blinded review of burn wound photographs by a panel of burn specialists. Burn wounds will be photographed by the ROs, using the study iPhone, at each dressing change until the wound is considered 95% re-epithelialised by the child’s treating clinician, and the wound no longer requires silver dressings. A panel of burn experts (comprising of pediatric surgical consultants and experienced burn nurses) will perform a blinded review of the patient’s photographed burn wounds–assessing if wounds are 95% re-epithelialised, or not. This method has previously been used in recent trials assessing time to re-epithelialization in pediatric burn patients [60–63].

#### Grafting and scar management

The proportion of pediatric burn patients who require a skin graft for their injuries (rate of skin grafting), as well as referrals for scar management, and adverse events (AEs), will be documented in this investigation.

#### Biological markers of inflammation and healing

Biological molecules associated with healing (e.g., inflammatory markers, extracellular matrix production or remodeling, skin cell growth) will be assessed using blood plasma, wound exudate, and urine biospecimens. Proteins and metabolites present within the samples will be measured using Mass Spectrometry techniques, as has been conducted previously [32,56,59,64]. Comprehensive mass spectrometry analysis is feasible from biological samples collected on paper [65]. Additionally, inflammatory cytokines and chemokines in plasma will be assessed using multiplex assays [66]. The levels of the biological markers will be related to the healing outcomes to identify biological and biochemical mechanisms associated with wound healing [56].

#### Stress

Biological markers of stress will be examined in hair, urine, and blood serum/plasma. Mass spectrometry and immunoassays will be used to quantify cortisol (in hair, urine and blood) [67,68], catecholamines (in urine and blood), and pro-inflammatory cytokines in blood. The measurement of cortisol and other stress-related markers in pediatric burn patients will provide insight into the impact of the burn on a range of body systems–and how this impact might lead to prolonged wound healing and maladaptive outcomes in children with burn injuries. Moreover, there is concern among clinicians that NPWT will increase burden and distress for pediatric burn patients and their families, so as part of this investigation, biological markers of stress will be examined in children treated with and without adjunctive NPWT for their acute burn injuries.

#### Operating theatre requirements

The proportion (percentage) of patients requiring an operation, in addition to the number of operations required per patient, and the type of operative procedures performed (e.g., skin graft) will be collected via observation and/or EMR assessment. The number and type of dressing changes until wound healing will also be recorded for all patients via observation and EMR assessment.

#### Hospital requirements 12 months post-injury

Rate of patients needing ≥1 admissions to hospital (% and length of stay), as well as number of outpatient appointments and number of scar clinic appointments will be recorded via observation and electronic medical record assessment up to 12 months post-injury.

#### Healthcare resource use for study interventions

Healthcare resource use will include application and removal of NPWT, device costs, other relevant dressings, procedures, and unexpected return to hospital. This data will be captured via observation and review of EMRs.

## Statistical analysis

### Sample size and statistical power

Using a SW-RCT design and four sites with a minimum of 100 individuals recruited from each site (recruiting patients until the site’s capacity is reached), we would be able to detect a difference of 10% in adoption rate with 94% power (5% alpha). We expect an improvement of 10% in adoption from 10% in controls to 20% in the treatment. A greater difference in adoption, with the same parameters otherwise, will result in a higher power, and thus, the above is the minimum number expected. Calculations were performed with the shiny app from Hemming *et al*. (2015) [45]. We anticipate a minimum of 120 potential patients per site, which will exceed the above estimate; thus, the power of this study is expected to be >94%.

### Statistical analysis plan

For quantitative measurements, appropriate regression models will be the main analyses. These will include simple linear regression, binary logistic regression, and Poisson/negative binomial regressions for continuous, binary, and count data, respectively. Both the parametric and bootstrapped versions will be implemented in order to get the most robust estimates from the data collected. Furthermore, Generalized Linear Mixed Models and Generalized Estimating Equations with appropriate assumed distributions as above will be used wherever warranted, especially to account for repeated measures within patient and cluster effects. An overview of the use of mixed models in stepped wedge trials with mixed models is given by Li et al. (2021) [69]. In this case an appropriate protocol for model selection based on Zuur et al. (2009) will be applied [70]. This analysis approach accounts for potential temporal trends, using time as a covariate, in addition to clinical explanatory variables (TBSA, age, gender), which will be included in each model. It should be noted that other covariates, such as time, could be used as random effects in the mixed models. For time-to-event outcome variables, such as time to re-epithelization, a time to event analysis in the form of Cox-proportional hazards regression will be employed. In every instance, appropriate summary statistics and exploratory analyses will be applied to get a full understanding of the data at hand.

### Cost-effectiveness analysis

We will record data, analyze, and report cost-effectiveness findings following guidelines for trial-based cost-effectiveness analyses [71], including reporting resource use and costs (healthcare perspective) for each trial condition. When available, healthcare utilization data will be costed using actual costs (e.g., device costings) or market rates. Intervention provision costs to deliver usual care or acute adjunctive NPWT in parallel with the INPREP toolkit during the trial (12-month time-horizon for patients) will be recorded and applied at a per-patient level. If appropriate, a trial-based incremental cost-effectiveness ratio (ICER) will be estimated for the incremental cost per additional patient successfully receiving acute adjunctive NPWT in parallel with the INPREP toolkit. ICER = [(CostNPWT) minus (Costusual care)] / [(EffectNPWT minus Effectusual care)]. Due to the potential for uncertainty and non-normal distributions, 95%CIs (for costs and effect estimates) and a 95% confidence ellipse (for ICER) will be derived from bootstrap resampling. If differences between groups remain at the primary time-horizon, extrapolation modelling will be considered to extend estimates to longer time-horizons that may be relevant to study stakeholders (e.g., 5-year time-horizon).

## Discussion

Given existing levels of morbidity among children with burns, innovative, cost-effective, and sustainable solutions are required to decrease healing times and reduce the risk of scarring. Evidence generated by this trial will inform pediatric burn care internationally. Clinical practice guidelines in Australia supporting the application of adjunctive NPWT in acute burn wound management are currently not clearly defined or easily accessible. Moreover, the use of NPWT in burns varies across Australian pediatric burn services. At present, Australian pediatric burn services commonly use NPWT as a bolster dressing following skin grafting procedures to promote graft take and reduce sheering forces. Whilst several pediatric burn services in Australia have made ad hoc or selective use of adjunctive NPWT in acute burn care, only one has adopted NPWT as part of routine burn care for acute injuries to date.

### Trial status

This manuscript outlines a protocol for a SW-RCT: protocol version number 3.0 version, date 9^th^ June 2023. Recruitment for this SW-RCT commenced on 3 July 2023 and will continue until September 2024.

## Supporting information

S1 Protocol (DOCX)

S1 File Negative pressure wound therapy decision pathway (master version). (DOCX)

S2 File Negative pressure wound therapy clinician handout. (DOCX)

S3 File Negative pressure wound therapy parent-caregiver education handout. (DOCX)

S4 File Wound fluid collection, processing, and storage. (DOCX)

S5 File Blood collection, processing, and storage. (DOCX)

S6 File Hair collection, processing and storage. (DOCX)

S7 File Urine collection, processing and storage. (DOCX)

## Abbreviations

AE: Adverse Events
ANZCTR: Australian and New Zealand Clinical Trials Registry
BRANZ: Burns Registry of Australian and New Zealand
CFIR: Consolidated Framework for Implementation Research
CI: Confidence Interval
COD: Change of Dressing
CTN: Clinical Trials Notification
ED: Emergency Department
EMR: Electronic Medical Records
GA: General Anesthetic
GCP: Good Clinical Practice
HREC: Human Research Ethics Committee
ICER: Incremental Cost-Effectiveness Ratio
INPREP Toolkit: Implementation of NPWT For Acute Pediatric Burn Injuries Toolkit
MS: Mass Spectrometry
NPWT: Negative Pressure Wound Therapy
PBMC: Peripheral Blood Mononuclear Cells
RCT: Randomized Controlled Trial
REDCap: Research Electronic Data Capture
ROs: Research Officers
SW-RCT: Stepped-Wedge Randomized Controlled Trial
TBSA: Total Body Surface Area
