# Supplementary material for: Implementation of negative pressure for acute pediatric burns (INPREP): A stepped-wedge cluster randomized controlled trial protocol
Source: PLoS One. 2024 Dec 10;19(12):e0315278. doi: 10.1371/journal.pone.0315278 (PMC11630585; doi:10.1371/journal.pone.0315278)
Supplement: S1 Protocol — (DOCX) [file pone.0315278.s001.docx]

**IMPLEMENTATION OF NEGATIVE PRESSURE FOR ACUTE PAEDIATRIC BURNS**

**Version: 3.0**

**Date: 09/06/2023**

**Statement of Compliance**

**This study will be conducted in compliance with all stipulation of this protocol, the conditions of the ethics committee approval, the NHMRC National Statement on Ethical Conduct in Human Research and the Note for Good Clinical Practice.**

**STUDY INVESTIGATOR(S)**

Chief Investigator (A): Associate Professor Bronwyn Griffin^1^

Chief Investigator (B): Professor Roy Kimble^2, 10^

Chief Investigator (C): Professor Fiona Wood^3^

Chief Investigator (D): Professor Andrew Holland^4^

Chief Investigator (E): Professor Steven McPhail^6^

Chief Investigator (F): Professor Warwick Teague^7^

Chief Investigator (G): Associate Professor Leila Cuttle^5^

Chief Investigator (H): Professor Jed Duff^5^

Chief Investigator (I): Dr Natalie Philips^2^

Chief Investigator (C): Associate Professor Dianne Crellin^7, 8^

Co-Investigator (A): Ms Yvonne Singer^9^

Co-Investigator (B): Dr Cody Frear^10^

Co-Investigator (C): Associate Professor Dimitrios Vagenas^5^

Co-Investigator (D): Dr Alexandra De Young^2^

Co-Investigator (E): Associate Professor Pauline Calleja^11^

1: Menzies Health Institute Queensland, Griffith University, Nathan Campus, QLD, Australia 4111.

2: Children’s Health Queensland Hospital and Health Service, 501 Stanley Street, South Brisbane, QLD, Australia, 4101.

3: Burn Injury Research Unit, University of Western Australia, 35 Stirling Highway, Crawley, WA, Australia, 6009

4: Discipline of Paediatrics and Child Health, Sydney Medical School, Faculty of Medicine and Health, The University of Sydney, 3 Parramatta Road, Camperdown, NSW, Australia, 2050

5: School of Biomedical Sciences, Faculty of Health, Queensland University of Technology

6: Faculty of Health, School of Public health and Social Science, Queensland University of Technology

7: Murdoch Children’s Research Institute, Royal Children’s Hospital, 50 Flemington Road, Parkville, VIC, Australia 3052

8: Royal Children’s Hospital Melbourne, 50 Flemington Road, Parkville, VIC, Australia 3052

9: Victorian Adult Burn Service, Alfred Hospital, 55 Commercial Road, Melbourne, VIC, Australia 3004

10: Faculty of Medicine, University of Queensland, Herston, Brisbane, QLD, Australia 4006

11: School of Nursing, Midwifery & Social Sciences, Central Queensland University, Cairns, Australia 4870

**CORRESPONDING INVESTIGATOR**

Associate Professor Bronwyn Griffin

Level 7, Centre for Children’s Health Research

Graham St, South Brisbane QLD 4101

# Administrative Information

**TRIAL REGISTRATION**

Australian and New Zealand Clinical Trials Registry ID: ACTRN12622000166774

**HREC APPROVALS**

HREC/2021/QCHQ/81002

**FUNDING**

This project has been awarded funding from the National Health and Medical Research Council (NHMRC) Partnership Projects PRC2 Funding ID: 2006970.

**STATEMENT OF COMPLIANCE**

This document is a protocol for a clinical research study. The study will be conducted in compliance with all stipulations of this protocol, the conditions of ethics committee approval, the NHMRC National Statement on Ethical Conduct in Human Research (2007) and the Note for Guidance on Good Clinical Practice (CPMP/ICH-135/95).

**1. INTRODUCTION**

Each year thousands of children present to Australian Emergency Departments (EDs) with an acute burn injury. Childhood burns contribute substantially to the non-fatal burden of disease (1). Between 16 – 35% of paediatric burns patients develop hypertrophic scarring (2-7), which may impose long-term physical, cosmetic, and psychosocial burdens (2). Children with burns deserve high-quality treatment that can reduce the risk of these long-term sequelae (3). A critical goal of burn care is to achieve rapid healing of the burn. Time to re-epithelialisation is the most significant predictor for the development of hypertrophic scarring (7). Despite advances with the introduction of treatments including evidence-based first aid and silver-impregnated dressings (8-12), serious risks of poor long-term outcomes for children remain. Any interventions improving time to re-epithelialisation deserve serious consideration for incorporation into standard burn care.

Negative pressure wound therapy (NPWT) is a wound dressing system that provides sub-atmospheric pressure within a closed dressing. Evidence demonstrates that, when compared to standard treatment, the early application of NPWT to paediatric burns results in significant improvement in time to re-epithelialisation, with corresponding reduction sin the need for scar management and operating theatre time (13-17). Moreover, the total healthcare costs for treatment with NPWT were significantly lower (mean $903.69) per child when compared to standard silver dressings alone (mean $1,669.01) (13). The demonstrated clinical and cost efficacy of NPWT calls for widespread implementation. Despite the demonstrated clinical efficacy and cost-effectiveness of NPWT, evidence-based guidelines for its incorporation into acute paediatric burn care have not yet been devised.

We propose to facilitate the early implementation of NPWT into ED burn care practice to provide Australian children access to evidence-based treatment that may improve clinical outcomes and reduce healthcare costs. Partnering with Australia’s four major paediatric burns centres (The Children’s Hospital at Westmead, Perth Children’s Hospital, Queensland Children’s Hospital and Royal Children’s Hospital, Melbourne), we will co-design implementation strategies tailored to the complex acute burn care setting and evaluate the effectiveness of implementation. We hypothesise that the facilitated implementation will result in better adherence to early NPWT application in acute burn care settings (ED/burns centres). Leveraging previous, successful collaborations and international expertise of the CI team, results from this study will transform policy and practice to improve outcomes for Australian children.

**2. BACKGROUND**

In Australia, burns are one of the most common forms of childhood injuries, carrying significant patient morbidity (18). Scald and contact injuries comprise the greatest proportion of paediatric burns– often resulting from accidental hot liquid and food spills, and contact with hot surfaces (19). A significant proportion of childhood burns are small to medium sized. In accordance with recent reports from the Burns Registry of Australian and New Zealand (BRANZ), just under 90% of paediatric burn injuries were less than 10% total body surface area (TBSA). A burn less than 5% TBSA was recorded in 65% of paediatric cases (19). The majority of paediatric burns in Australia are small to median sized, however these injuries demand carefully planned treatment to reduce the risk of infection and improve time to reepithelialisation (20). In addition, despite significant advances in the treatment of burn injuries (i.e., fluid resuscitation, antimicrobial dressings, early excisional debridement), infection and scarring still remain common problems for burn patients (21, 22).Infection can lead to impaired and prolonged wound healing (23). Delays in healing time have been shown to significantly increase the child’s risk of developing hypertrophic scarring (2), which affects between 16% (4) and 35% (6) of children who sustain burns.

**2.1 Current wound therapy**

Compared to previous therapies, requiring inpatient management for daily dressings and higher rates of skin grafting, current therapies such as silver-impregnated multi-day dressings have seen admission and skin grafting rates decline. Despite these advances, children are still at risk of serious adverse outcomes that can impact long-term outcomes. Such outcomes may require multiple ongoing additional treatments, compounding the financial burden for families and the health care system, who provide the additional specialist time taken, extra scar therapy, and multiple operations required when healing time is delayed. Despite a relative paucity of economic evaluations in burns in Australia, it is accepted that they are one of the most expensive cohorts of patients in healthcare (24).

**2.2 Negative pressure wound therapy**

There is now strong evidence through experimental (16, 25, 26) and prospective cohort studies (15, 17, 27-29) that support the benefits of NPWT in acute burn care. Compared to standard silver dressings alone, adjunctive NPWT improves time to healing and decreases dressing change requirements and referrals for scar management. Negative pressure wound therapy (NPWT) offers a cost-effective solution for the treatment of paediatric burns. A recent study by our team evaluating the healthcare costs of NPWT in small-area paediatric burns found the mean total cost per person for the standard silver dressings group was $1,669.01 (95% CI 659.06 to 3269.16), compared to $903.69 (95% CI 670.68 to 1234.74) for the NPWT group (13).

**2.3 Implementation Challenge**

In Australia, clinical guidelines supporting the application of NPWT in burn wound management are currently not clearly defined or easily accessible. Moreover, the use of NPWT in burns is varied across Australian paediatric burns services and when it is used, it is purported to be challenging when attempting to optimise ambulation. In a recent investigation into the use and effectiveness of NPWT in a paediatric burn population, parents reported higher levels of treatment burden in patients who received adjunctive NPWT compared to those who received silver dressings alone. While adjunctive NWPT improved time to reepithelialisation and reduced referrals to scar management – NPWT was reported to limit movement and ambulation in young children (26).

The unique health service contexts of each state will require flexibility in pathway development. The key implementation challenge will be to co-design a new practice pathway that responds to the contextual characteristics of each site (The Children’s Hospital at Westmead, Perth Children’s Hospital, Queensland Children’s Hospital, and Royal Children’s Hospital Melbourne), whilst maintaining best practice. To this end, we will use the Consolidated Framework for Implementation Research (CFIR) (30), a tested implementation framework best suited to complex implementations. Use of this framework will assist us to determine barriers and enablers influencing behaviour, practice, and policy change requirements that will improve long-term sustainability. Without focused implementation, adoption of the **I**mplementation of **N**egative **P**ressu**R**e for acut**E** **P**aediatric burns (INPREP) Pathway and its benefits for paediatric burns patients will be further delayed.

| **The research team have conducted a series of studies, the findings of which** supports the components and implementation of this work (13, 26, 31).   - **We demonstrated the efficacy of the adjunctive use of NPWT for acute paediatric burns** in a RCT at Queensland Children’s Hospital (n=114) (26). When incorporated with the use of standard silver impregnated dressings, NPWT **improved time to wound closure** by 22% (95% CI 7-34, p=0.005). Patients who received NPWT had a 60% (95% CI 18-81, p=0.013) **reduced risk of long-term scar management** referral. A post hoc subgroup analysis suggests NPWT applied within 48 hours of the burn injury may provide even greater therapeutic benefits. - **We conducted a trial-based economic evaluation** from the healthcare provider perspective, prospectively recording resource usage and costs from the first visit to six-months following injury. Total mean healthcare costs for children who received **NPWT** **were lower** than standard silver dressings ($903.69 vs $1669.01) (13).   **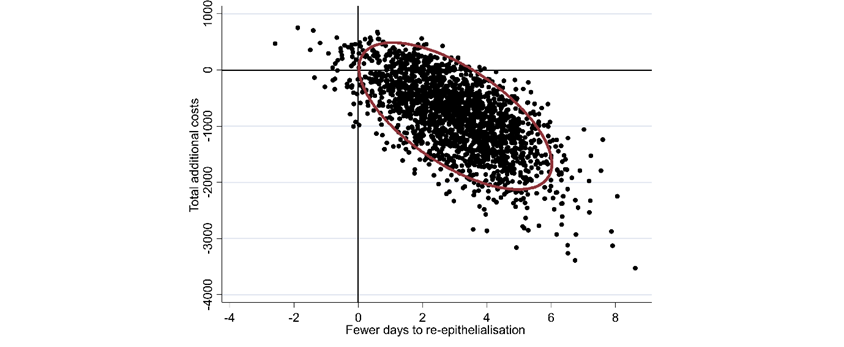**   - The greatest reductions were **related to scar management and operative interventions**. Primary analysis indicated that there was an **89% probability** **that** **NPWT was dominant** (lower costs and faster wound healing) (Figure 1). - **We were the first to characterise the proteome of wound fluid collected via NPWT (31)**. We identified and quantified the NPWT fluid constituent proteins using liquid chromatography tandem mass spectrometry and compared them to the proteome of matched paediatric burn wound exudate samples. Several proteins were more abundant in the NPWT fluid, including proteases and immune/inflammatory proteins. The results **support the hypothesis that NPWT augments wound healing through the modulation** of factors involved in the inflammatory response, granulation tissue synthesis, and extracellular matrix maintenance (31). More samples need to be collected to comprehensively understand the biological mechanism behind the effectiveness of NPWT.   ***Figure 1*** *Incremental cost-effectiveness of adjunctive NPWT versus standard care with 95% confidence ellipse* |
| --- |

**3. AIM OF STUDY**

The aim of this investigation is to examine the effectiveness of the INPREP Pathway and improve patient outcomes for children presenting to hospital with acute burn injuries.

**4. OBJECTIVES**

To achieve this aim, the following research objectives will be completed:

1. Co-design the adaption of an INPREP Pathway with clinicians (ED/Burns, nursing/medical) and consumers (patient and parent) to facilitate timely application of NPWT in acute paediatric burns.
2. Using a hybrid type III design, test the effectiveness of the INPREP Pathway implementation using (i) implementation outcomes (e.g., adherence, feasibility, sustainability) and (ii) clinical outcomes (e.g., days to re-epithelialisation, scar requirements, skin grafting requirements).
3. Disseminate recommendations to relevant organisations to inform policy development according to specific states and contexts.

**5. HYPOTHESIS**

**5.1 Primary Hypothesis**

Primary hypothesis: Our co-designed INPREP Pathway and implementation strategies will improve clinician adherence rates of NPWT application for the treatment of paediatric burns. This will also be considered acceptable, appropriate and feasible to clinicians, patients and carers alike.

**5.2 Secondary Hypotheses**

Secondary hypothesis: Paediatric patients with burns treated in the implementation phase will heal significantly faster resulting in reduced surgical requirements, scar treatment referrals and overall healthcare costs per patient compared to those treated in the control phase.

**6. STUDY DESIGN**

This project is a collaboration involving burns and ED clinicians from The Children’s Hospital at Westmead (CHW), Perth Children’s Hospital (PCH), Queensland Children’s Hospital (QCH) and Royal Children’s Hospital Melbourne (RCH). Conducted over three phases, the project will be underpinned by the tested implementation framework, associated measures, and evaluations. For ease of comprehension, research methods outlined in this protocol document have been separated into the three following project phases:

- **Phase 1** – Development of the INPREP Pathway using co-design principles; development of a baseline model of acute paediatric burn injuries in Australia; development of tailored implementation strategies for INPREP Pathway using implementation science frameworks.
- **Phase 2** – Test the implementation of the INPREP Pathway using a multi-centre, prospective, pragmatic, stepped-wedge randomised controlled trial (RCT).
- **Phase 3** – Develop clinical guidelines and implementation recommendations for each site and disseminate via relevant professional associations for local and regional pathway adaption.

**7. PHASE 1 RESEARCH METHODOLOGY**

**Phase 1. INPREP Pathway Co-design and associated implementation strategies**

The Consolidated Framework for Implementation Research (CFIR) will be used to inform the planning and evaluation of the INPREP Pathway and implementation strategies (30). The CFIR uses five domains (intervention, individuals, inner and outer setting, and the implementation process) to understand barriers and facilitators to implementation in complex systems. Leveraging the strength of the partnerships, the CFIR will be used to develop a comprehensive understanding of the complex contextual characteristics of acute burn care (ED/burns centres), across multiple contexts. This understanding will influence the implementation of the INPREP Pathway into clinical practice so that tailored implementation strategies can be better targeted. Implementation strategies will be selected from the Expert Recommendations for Implementing Change (ERIC). Identified CFIR barriers will be matched to implementation strategies using the CFIR-ERIC matching tool as well as the ERIC implementation strategies (32).

The NPWT Advisory Group (NAG) has been established to develop this proposal, consisting of investigators, clinical and patient/parent end users, and national professional organisations such as the Australia New Zealand Burns Association (ANZBA), Australasian College of Emergency Medicine (ACEM), Don’t Forget the Bubbles (DFTB), and the Paediatric Research Emergency Departments Internal Collaborative (PREDICT). The NAG are responsible for project oversight and strategic direction, ensuring engagement of their respective stakeholder organizations including identified local champions (to influence practice) and executives (to influence policy). The NAG influences the CFIR inner and outer settings of the INPREP Pathway implementation. They are uniquely placed to mobilise knowledge through organisations, influence behaviour, remove barriers and provide resources to overcome decision-making delays (33, 34). The NAG will hold bi-monthly webinars throughout project.

Our INPREP pathway co-design will allow clinicians to integrate NPWT into their model of care by considering important contextual factors. These factors will include:

1. Who applies the dressing – ED and/or burns clinicians?
2. When and where is the dressing applied – ED, burns department, or operating theatre?
3. How to apply the dressing?

We aim for a standard pathway, however contextualisation may be needed. For example, existing state burns models of care may require burns patients to have all dressings applied by burns staff, therefore bypassing ED clinicians.

**7.1 STUDY SETTING/ LOCATION**

Parent/caregivers, as well as burns and ED clinicians from the four partnering sites, will complete electronic surveys and participate in phone or onsite focus group interviews. The surveys will be completed online in their own time and in an environment of their choice. Phone interviews will be scheduled to suit the parent, caregiver, or clinicians and co-design focus group will be conducted on site at the Centre for Children’s Health Research in South Brisbane, with COVID-19 safe approaches.

- 1. **STUDY POPULATION**
     1. **Burns and Emergency Department Clinicians (nursing and medical)**

This group will interact with the implementation through surveys and interviews in all phases of the study. Clinicians will be sampled from The Children’s Hospital at Westmead (CHW), Perth Children’s Hospital (PCH), Queensland Children’s Hospital (QCH), and Royal Children’s Hospital Melbourne (RCH).

- - 1. **Parents and caregivers of children who have engaged in the above burns services**

This group will inform the NPWT advisory group (NAG) in Phase 1 of this investigation and provide consumer outcomes. Parents and caregiver of paediatric burn patients referred to, or engaged with, the Burns Service at the CHW, PCH, QCH, or RCH will comprise this population.

**7.3 ELIGIBILITY CRITERIA**

**7.3.1. Inclusion criteria**

- Clinicians from the ED and/or burns service who are responsible for burns care
- ED and burns clinicians from regional and/or remote settings who are responsible for burns care
- Parents and caregivers of children who have had a burn injury within the last year

**7.3.2. Exclusion criteria**

- Clinicians who are not currently working in a clinical capacity within the ED or Burns Service
- Parents and caregivers of paediatric patients >1 years since burn injury.

**7.4 STUDY OUTCOMES**

**7.4.1 Primary Outcome**

1. Co-development of an agreed INPREP Pathway and complimentary implementation strategies

**7.4.2 Secondary Outcomes**

1. Define baseline model of care for paediatric burn injuries across the four tertiary burns centres
2. Identify perceived barriers and enablers to introducing NPWT and the INPREP Pathway
3. Reach consensus with key stakeholders regarding the use of NPWT in acute paediatric burn patients

**7.5 STUDY PROCEDURES**

**7.5.1 Recruitment of participants**

Key ***clinical stakeholders*** who represent multidisciplinary paediatric burns and emergency clinicians will be identified by local CIs and approached by local Research Nurse (ReN) and delivered a participant information and consent form (PICF) prior to participation in either ***i) interview, ii) focus group and/or iii) questionnaire***. This will outline the basic structure of the key questions and discussions to be held.

Key ***parent/carer stakeholders*** will be identified by the local burns services. Once parent and caregiver stakeholders are identified as potential participants, the Research Nurse (ReN) will give carers an opportunity to read through the PICF and collect consent after the adequate opportunity to peruse and ask questions. We will cease recruitment once we have successfully recruited up to 8 participants.

At the time of recruitment clinician and parent/carer participants will be informed that focus groups and interviews will be flexible to face to face, telephone, or web-based video conference to accommodate participant preference. All stakeholders will be given the opportunity to view their personalised coded data – in case they wish to retract, review, or edit their responses prior to publication. Due to the risk of COVID – 19 travel restrictions we have decided to minimise travel interstate and participants will be restricted to web-based video conference only.

**7.5.2 Sampling and data analysis**

Co-design of the INPREP Pathway will be informed by Negative pressure Advisory Group (NAG) interviews and questionnaires of specialty groups – including ANZBA, ACEM, DFTB, and PREDICT.

1. **NAG Stakeholder interviews**

Semi-structured interviews will be conducted using maximum variation sampling (until saturation is reached) to ensure representation of different perspectives including emergency and burns clinicians (medical/nursing, metropolitan/regional, novice/experienced, various states) and consumers (parents/carers of NPWT patients) across all states. Interviews will be audio recorded using a semi-structured approach with open-ended questions about current and desired approaches to NPWT application in paediatric burns patients. Interviews will aim to identify contextual barriers to and enablers of the use of NPWT. If interviewees refer to related documents or tools, we will collect these. Regarding data analysis, transcribed interviews will be analysed using a deductive content analysis procedure recommended for CFIR (35). The analysis will be performed by two researchers independently using the CFIR NVivo template pre-populated with construct codes, with the ability to add new intervention codes where relevant. NVivo is a qualitative data analysis computer software package produced by QSR International [QSR International Pty Ltd. (2020) NVivo (released in March 2020) [https://www.qsrinternational.com/nvivo-qualitative-data-analysis-software/home](https://www.qsrinternational.com/nvivo-qualitative-data-analysis-software/home?_ga=2.227843295.371367470.1635126007-457000355.1635126007)]. Coded text will then be subjected to a rating process following CFIR recommendations (30). In the rating process, a deliberated consensus process will be used to assign a rating that reflects the positive or negative influence and the magnitude or strength of each construct.

1. **Supplementary web-based questionnaires**

A questionnaire built on the CFIR constructs will identify barriers to and enablers of the implementation of the INPREP Pathway in settings other than the four study sites. Questionnaires will be distributed via existing network pathways including ANZBA, ACEM, DFTB, PREDICT, and Rural and Remote Health. Findings from the supplementary web-based questionnaires and the NAG stakeholder interviews will be used to triangulate data and inform the consensus focus group.

1. **NAG Consensus Focus Group**

To develop implementation strategies, the NAG, consisting of researchers, clinician and parent/carers (n=) will consider identified contextual barriers and enablers identified through key stakeholder interviews, and questionnaires, underpinned by the CFIR. These factors will be mapped to the Expert Recommendations for Implementing Change (ERIC) taxonomy, using a tested tool that provides a prioritised list of strategies to consider based on identified CFIR factors (36). For example, the CFIR-ERIC tool will provide education and training strategies for the identified knowledge deficit, which may need to be adapted based on context). This will guide evidence-based decisions about strategies that may influence INPREP Pathway adoption and implementation. The NAG will convene as a group via web meetings to confirm appropriate strategies for the INPREP Pathway implementation components and plan.

**8. PHASE 2 RESEARCH METHODOLOGY**

**Phase 2. TEST THE INPREP PATHWAY EFFECT ON IMPLEMENTATION AND CLINICAL OUTCOMES**

Following the development and co-design of the INPREP Pathway, NPWT will be implemented and integrated across four major paediatric Australian hospitals using a hybrid type III effectiveness-implementation design to test the INPREP Pathway implementation strategies and patient outcomes, via a multi-centre, prospective, pragmatic, stepped-wedge RCT at four partnering hospitals. This hybrid design is highly suited to evaluate service delivery interventions where evidence exists, and partnership is strong (37). The stepped-wedge RCT suits scenarios where simultaneous rollout is unfeasible (38). We aim to evaluate the impact of the INPREP Pathway on implementation outcomes and patient outcomes over three periods (control, implementation, and sustainability – see Figure 2 below).

**8.1 STUDY SETTING/LOCATION**

Phase 2 of this investigation will be conducted across four Australian paediatric burn centres within the ED, operating theatre, and burns departments. Site locations will include the following:

1. The Children’s Hospital at Westmead (CHW)

The Children's Hospital at Westmead is the largest paediatric centre in New South Wales, and provides tertiary specialist medical care for children across New South Wales, greater Australia. The CHW houses the states paediatric burns centre treating more than 800 paediatric burns patients per annum (p.a).

1. Perth Children’s Hospital (PCH)

Perth Children's Hospital is a public tertiary children's hospital located in Nedlands, Western Australia. It is Western Australia’s largest paediatric hospital and trauma centre – providing specialist medical care to children under the age of fifteen years. The PCH houses the state’s paediatric burns centre treating more than 700 paediatric burns patients p.a.

1. Queensland Children’s Hospital (QCH)

The Queensland Children's Hospital is a public tertiary children's hospital in South Brisbane, Queensland, Australia. It is the single specialist paediatric hospital for Queensland, with a catchment area extending to northern New South Wales. The QCH houses the states paediatric burns centre treating over 1000 burn injuries in children p.a.

1. Royal Children’s Hospital Melbourne (RCHM)

The Royal Children's Hospital is a public tertiary children's hospital in Melbourne, Australia. It is the major specialist paediatric hospital and trauma centre in Victoria, with a catchment extending to children from Tasmania, southern New South Wales, and other states around Australia and overseas. The RCHM houses the states paediatric burns centre treating over 500 paediatric burn patients p.a.

- 1. **STUDY POPULATION**

For Phase 2 of this investigation, participants will include paediatric patients (aged less than 18 years) presenting to the CHW, PCH, QCH, or RCHM with an acute burn. Specific eligibility criteria (inclusion and exclusion criteria) for the use of NPWT in children with acute burns will be established during Phase 1 of this investigation. Broad inclusion criteria will include children presenting to one of the four participating tertiary hospitals with an acute thermal burn injury requiring treatment from the Burns Service.

**8.3 STUDY OUTCOMES**

**8.3.1 Primary Outcome**

1. INPREP Pathway adherence: rate of patients who received NPWT (according to eligibility and duration of application defined within the INPREP Pathway)

**8.3.2 Secondary Outcomes**

***Secondary Implementation Outcomes:***

1. *Appropriateness* – clinician and patient carer perspective of pathway and procedure, Intervention Appropriateness Measure (39), CFIR observation/field notes
2. *Acceptability* clinician and patient carer perspective, Acceptability of Intervention Measure (39), and CFIR observation/field notes
3. *Feasibility* clinician and patient carer perspective, Feasibility of Intervention Measure (39), and CFIR observation/field notes

***Secondary Patient Outcomes:***

1. *Healing outcomes:* i) Time/days to re-epithelialisation (blinded photo assessment), ii) skin grafting requirements, iii) burn wound exudate, iv) number of dressing changes, v) adverse events (26).
2. *Operating theatre requirements:* i) proportion patients requiring an operation (%), ii) operations required per patient, iii) operative procedures performed (e.g., skin graft) (26).
3. *Hospital requirements 12 months post-injury:* i) proportion of patients needing ≥1 admissions to hospital (% & length of stay), ii) number of outpatient appointments, iii) number of scar clinic appointments.
4. *NPWT device malfunctions:* i) alarms ii) charging issues

***Secondary Resource use outcomes (12-month time horizon):***

1. Implementation costs (e.g., change facilitator, staff time for training, resources and materials).
2. Health care resource use for study interventions (e.g., time to apply NPWT, cost of device, other dressings used, procedures, unexpected return to hospital, time to remove and reapply NPWT at follow-up dressing change appointments)

**8.4 STUDY PROCEDURES**

Five steps will be sequentially rolled out across the 4 hospitals over 13 months: **1. Set up** ensures adequate training and collection of control and intervention data. **2. Control steps**-are usual care, i.e., standard silver dressings. **3.** **Intervention establishment** involves the research nurses (ReNs) collaborating with the NAG and local champions to promote the INPREP Pathway and delivering the companion implementation strategies. A project manager will develop a study manual and training schedule for ReNs, monitor data integrity, intervention fidelity log and collaborate with the team (ReNs and CIs) to promote intervention fidelity. **4. Implementation period** is intervention exposure. **5. Sustainability** of adherence measured. In both control and implementation steps other burn care will be as per local standards (40). Implementation education will provide evidence-based advice, but treating clinicians will determine definitive care (e.g., inpatient versus outpatient care) based on patient needs and healthcare priorities.

**Figure 2.** Steps for the multi-centre, prospective, pragmatic, stepped-wedged randomised controlled trial


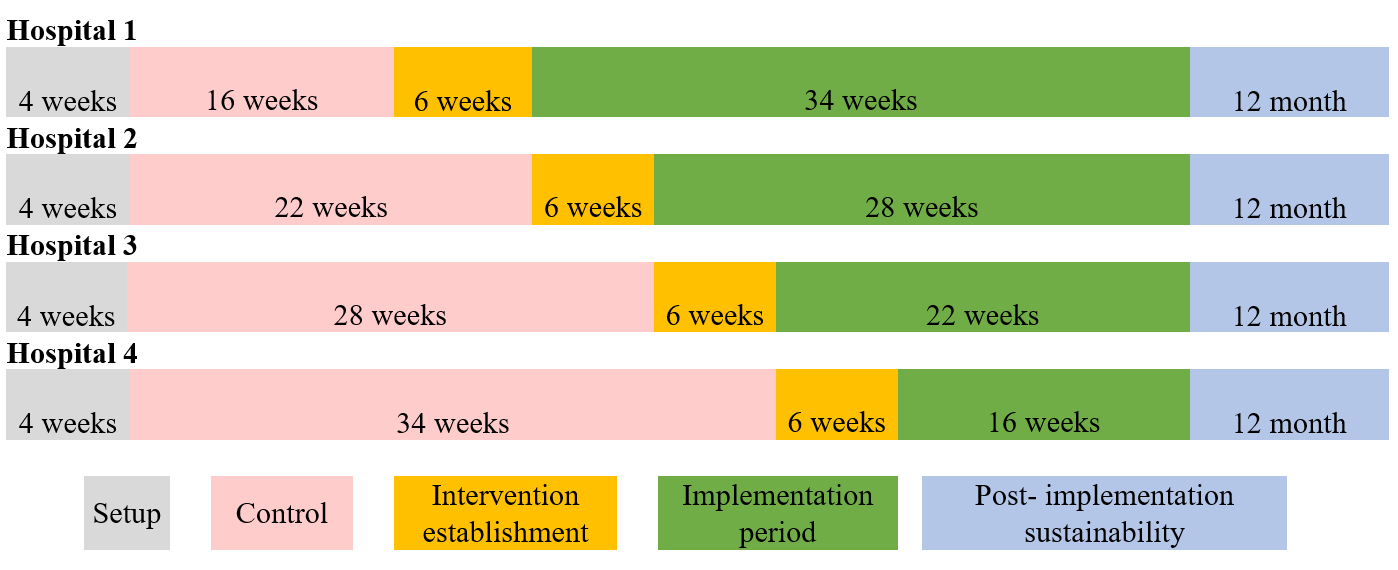


**Figure 2**. Illustrates the five steps of the multi-centre, prospective, pragmatic, stepped-wedged randomised controlled trial that will be conducted across the four participating paediatric hospitals. Following initial set up, all participating sites will start the trial in control phase with the baseline measures taken, and then one site will randomly step up (computer-generated, centralised randomisation) to implementation every 6 weeks, until saturation of the implementation intervention across all sites.

**8.4.1 Recruitment of participants**

Wherever possible, prospective informed consent will be obtained from parents and caregivers (and the child where age appropriate) in the ED following initial patient presentation to hospital. We are requesting consent to continue (also referred to as deferred consent or research without prior consent) for Phase 2 of this investigation in extenuating circumstances where prospective informed consent is not possible, practical, or appropriate to obtain. As the intervention in this trial is accepted as current clinical practice, and carries no greater risk than standard care, deferred consent will be obtained in certain circumstances where approaching the parent/caregiver and child in the ED is considered to be inappropriate, and likely to increase parental and patient distress. In these circumstances where deferred consent is required, parents and caregivers will be informed about the research as soon as possible after it commences and their written consent for continuing involvement will be sought. Consent to continue (deferred consent) will be obtained from parents and caregivers within 24-hours following initial presentation to hospital for their child’s burn injuries. Consent will be obtained as close to 24-hours as possible but no later than 72-hours. Parents and caregivers, and the child where age appropriate, will be approached by a member of the research team and presented with information regarding the trial in the ED. Parents and caregivers will be given an opportunity to read through the PICFs in the ED and any questions they have will be answered. In cases where the emergency situation surrounding the child or young person is sufficiently distressing to the parent or carer, and it could be considered inappropriate to approach the parents acutely, verbal consent will be obtained via telephone within 24 hours after commencement of the research. Following verbal telephone consent, written consent for continuing involvement will be obtained at the child’s first dressing change in the Burns Outpatient department for paediatric outpatients, and within 24-hours for inpatients.

For paediatric patients and families not scheduled to return to the Burns Outpatient Department within 72 hours post-burn, written informed consent will be obtained using a REDCap link tick box – emailed to the parents and carers. It will be made clear that if the parent/caregiver withdraws at that point, or anytime thereafter, they will be given the option to have any collected data or samples destroyed. All communications between research staff and parents and caregivers will account for, and take into consideration, the stress and emotional factors associated with having an acutely burned child. Research staff will ensure that the dependency of potential participants and their patents and caregivers on ED and burns clinicians providing burn treatment will not influence their decision to participant in the research. Paediatric patients involved in this investigation may be critically ill, and unfortunately despite best efforts, some of the patients may die as a result of their burn injury before permission to use the collected data can be obtained. In these cases, data will be discarded and all biospecimen samples will be destroyed as per standard operating procedures for the destruction of biological samples at each partnering site.

**8.4.2 Data Collection**

Research Nurses (ReNs) will collect primary and secondary outcome data prospectively by a combination of observation of, clinical staff, and patients/carers and electronic medical records (EMRs), using methods from previous studies (14, 41). Patient demographic and clinical characteristics will be recorded at initial presentation to the ED or burns service via EMR. Wound photos will be taken with a study iPad at each dressing change and deidentified for blinded review to assess and establish time to healing. ReNs will collect hospital data interactions (such as outpatient appointments, planned or unplanned return to ED, operating theatre and admission requirements) from each patient for 12 months post injury. Where possible burn wound exudate will be collected at NPWT application and removal and will be analysed using previous techniques (31) and related to the healing outcomes, to identify biological and biochemical mechanisms (42). ReNs will capture site level measures (e.g., ratios of burns treatment complications). The schedule of assessments for the trial is presented in Appendix 5.

Data will be directly entered by ReNs into a REDCap database (Research Electronic Data Capture, Vanderbilt, USA, hosted at Griffith University) using password-protected iPads. Identifying information will only be stored at the site level. Patient questionnaires will be completed over the telephone by an alternative site ReN (who is blinded to the crossover schedules) at three months post burn injury. We will instruct patients not to disclose their allocation (control or INPREP) to the ReN. Staff web-based questionnaires will be sent out 4 weeks into implementation and at completion of implementation phase. ReNs will conduct CFIR-informed clinician observations, interviews, and document analysis at each participating site.

**8.4.3 Collection of Biospecimens**

Potential participants will be approached for biospecimen collection if it will be possible to collect a sample of their burn wound exudate, blood, or urine as part of their normal wound care procedures or standard care. As part of routine burn wound cleaning procedures, wound exudate or fluid is removed and usually discarded. Instead, for this study, it will be retained for analysis. If participants will be sedated or under a general anaesthetic (GA) for their standard wound care procedures, or require a cannula or routine blood draw at the time of their hospital visit, this will enable collection of blood, which will be retained for analysis. If participants are scheduled to receive a urinary catheter as part of routine standard treatment for their burns, this will enable the collection of urine that will be retained for analysis. For participants who do not require a catheter, the child and parent/caregiver will be asked if they consent to providing a urine sample and will be given a urine sample pot container. Whilst not collected as part of routine standard care for burn injuries, hair samples will also be collected from consenting patients and parents/caregivers. Additional information on biospecimen sampling is provided under section *8.4.4.2 Healing outcomes.*

- - 1. **Measurement tools used**
       1. Appropriateness, Acceptability, and Feasibility of the INPREP Pathway
- Appropriateness of the INPREP Pathway and procedures, from the perspective of the clinician and patient carer, will be assessed using the Intervention Appropriateness Measure (39) and CFIR observation and/or field notes
- Acceptability of the INPREP Pathway, from the perspective of the clinician and patient carer will be examined using the Acceptability of Intervention Measure (39) and CFIR observation and/or field notes
- Feasibility (from the clinician and patient carer perspective) of the INPREP Pathway will be assessed using the Feasibility of Intervention Measure (39) and CFIR observation/field notes
  - - 1. Healing outcomes
- Time to re-epithelialisation (days) will be examined using blinded photo assessment of patient’s injuries. Wounds will be photographed by the ReNs, using the study iPad, at each dressing change until the wound is considered 95% re-epithelialised by the child’s treating clinician at the site, and the wound no longer requires definitive silver dressings. A panel of burn experts (comprising of paediatric surgical consultants and experienced burns nurses) will perform a blinded review of patient’s photographed burn wounds – assessing if wounds are 95% re-epithelialised. Photos will be taken of patient’s burn injuries by the ReN at each follow up dressing change appointments This method has previously been used in recent trials assessing time to re-epithelialisation in paediatric burn patients (14, 41).
- Proportion of paediatric burn patients who require a skin graft for their injuries (rate of skin grafting), as well as adverse events (AE) and referrals to scar management will be documented in this investigation. The number, and type, of dressing changes until wound closure will also be recorded for all patients via observation and electronic medical record assessment.
- Biological molecules associated with healing (e.g., inflammatory markers, extracellular matrix production or remodelling, skin cell growth) will be assessed using wound exudate analysis. Any wound fluid able to be collected at NPWT application and removal will be analysed using previous techniques (31, 43) and related to the healing outcomes, to identify biological and biochemical mechanisms associated with wound healing or scarring (42).

*Wound Exudate Collection:*

Wound exudate will be collected from the participants during routine wound cleaning procedures. Instead of being discarded, the burn wound exudate will be retained and stored using the Standard Operating Procedure SOP-Wound-Fluid (see Appendix 1). Filter paper will be placed at the edge of the burn wound to absorb the wound exudate (44, 45). A comprehensive mass spectrometry (MS) analysis is possible from biological samples collected on paper (46). Researchers at the QCH site have previous experience collecting wound exudate from NPWT devices (43) and directly from the wound (47), and identifying proteins in these samples (31). Briefly, samples will be centrifuged to pellet cell debris and the supernatant will be aliquoted into tubes. Samples will be stored at -80C in a research facility or hospital laboratory at each site.

- Stress will be examined using a number of techniques in this investigation including hair, urine and blood, analysis where opportunistic sampling is possible – and when resources and ethics is available at partnering sites. The collection of blood and hair samples from paediatric burn patients will provide insight into the impact of the burn on a range of body systems – and how this impact might lead to prolonged wound healing and maladaptive outcomes in children with burn injuries. See Appendix 2 and 3 for additional methods and procedures for patient blood and hair sampling.

*Blood Collection:*

Using an opportunistic sampling method, blood will be collected from the participants during routine wound care procedures. In cases where the participant is scheduled to receive a routine blood draw, canula insertion, or is receiving a GA for routine burn treatment – blood will be collected, processed and stored using SOP-Blood-Processing (see Appendix 2). Briefly, blood will initially be collected in a heparinised tube. A small amount of whole blood will be removed and prepared for transcriptomic (gene expression), epigenetic and immune phenotypic assays. The remaining sample will be processed through several centrifugation steps, and Peripheral Blood Mononuclear Cells (PBMCs) and plasma will be isolated. The plasma, whole blood isolates and cells will be aliquoted into tubes. Plasma and whole blood samples will be stored at -80^◦^C and cells will be stored in liquid Nitrogen in a research facility or hospital laboratory at each site. No more than 5% of the whole blood volume will be removed in a single occasion. In cases where paediatric patients do not require blood sampling, canulisation, or a GA as part of their routine standard care – blood collection will not be performed. All patients will be offered a numbing cream to help reduce pain during blood sampling if there are concerns regarding discomfort.

*Hair Collection:*

A hair sample containing approximately 100 strands of hair and a minimum of 3cm in length will be collected from the area at the nape of the neck, cut as close to the scalp as possible using scissors (see Appendix 3). Patient’s hair characteristics (e.g. colour, washing episodes per week, and presence of hair dye or product) will also be recorded from parents and caregivers, and the child where age appropriate.

*Urine Collection:*

Where possible, urine will be collected from the participants. If participants are scheduled to receive a urinary catheter as part of routine standard treatment for their burns, some urine that will be retained for analysis (see Appendix 4). For participants who do not require a catheter, the child and parent/caregiver will be asked if they consent to providing a urine sample and will be given a urine sample pot container for collection. A minimum volume of 60mL is required for analysis. The sample will be immediately frozen at -20^◦^C to prevent microbial contamination or proteolytic degradation. The next day or later, the sample will be clarified by centrifugation, filtered to remove bacteria or protein aggregates, and concentrated using previously established techniques (48) and then stored at -80^◦^C in aliquots for analysis.

- - - 1. Operating theatre requirements
- Proportion (%) of patients requiring an operation, in addition to the number of operations required per patient, and the type of operative procedures performed (e.g., skin graft) will be recorded via observation and electronic medical record assessment
  - - 1. Hospital requirements 12 months post-injury
- Rate of patients needing ≥1 admissions to hospital (% and length of stay), as well as number of outpatient appointments and number of scar clinic appointments will be recorded via observation and electronic medical record assessment
  - - 1. Healthcare resource use for study interventions

Healthcare resource use will be calculated using variables such as time to apply NPWT, cost of device, other dressings, procedures, and unexpected return to hospital. This data will be captured via observation and review of electronic medical records

- - - 1. Implementation costs

Healthcare resource use will be calculated using variables including change facilitator, staff time for training, resources, and materials.

**9**. **PHASE 3 RESEARCH METHODOLOGY**

**PHASE 3. TRANSLATION OF INPREP RECOMMENDATIONS**

The NAG will review the findings of Phase 2 against the implementation strategies developed in Phase 1. The NAG will refine implementation strategies based on findings from Phases 1 and 2, and develop and distribute key findings and recommendations to update INPREP Pathways and clinical guidelines at state and national levels. The NAG will utilise the extensive professional national networks represented across our investigatory team to enhance dissemination of the refined implementation strategies. Graphic design experts will be engaged to enhance presentation of the material for distribution. Our key recommendations will aim to allow each organisation to adopt guidelines and policy for their own context.

Knowledge translation of NPWT resources will include:

1. Conference presentations
2. Open access peer-reviewed publications
3. Targeted social media postings (twitter)
4. Podcasts
5. Blog posts
6. Champion opinion leaders

To ensure national clinical guideline recommendations are reviewed for national acceptance, findings will be presented to the clinical excellence committees for ANZBA (CIs Kimble, Wood, Holland, and Teague), PREDICT Executive (CIs Griffin and Phillips), and CENA Clinical Excellence Committee (CIs Griffin and Crellin). Moreover, a workshop detailing clinical guidelines for NPWT use in paediatric burn patients will be planned for the ANZBA Annual Scientific Meeting. Other measures to disseminate research findings will include distributing educational materials including the INPREP Pathway and an implementation manual to guide behavioural change to all Australian paediatric burns centres. Lastly, we aim to develop a web-based/digital resources that can be made available to key stakeholders to promote findings and offer a decision-making algorithm for choosing implementation strategies to supplement the clinical guideline.

**10. DATA MONITORING**

REDCap data will be backed up on the Griffith University Data Storage Service. Participants will not be identified by name, and confidentiality of patient information will be preserved. All participants details will be entered in coded format and the confidentiality of the participant will be maintained unless disclosure is required by law. Access to the data collection form will only be available to necessary members of research team and stored in accordance with data storage best practices. Any data exported from REDCap will be exported without any identifiable information, and no identifiable information data will be published. Deidentified clinician and consumer data from both phases will be kept in a locked filling cabinet, behind swipe card access only doors at the Centre for Children’s Health Research in Brisbane. An annual data management plan will be reviewed and maintained by an investigator aligned with the study.

**10.1 Data management and storage**

Electronic patient data will be backed up on the Griffith University Data Storage Service. Deidentified clinician and consumer data from both phases will be kept in a locked filling cabinet, behind swipe card access only doors at the Centre for Children’s Health Research in South Brisbane. An annual data management plan will be reviewed and maintained by an investigator aligned with the study. In accordance with the National Health and Medical Research Council guidelines, all data and samples will be stored for 15 years after completion of the study, or until the youngest person taking part in the study is 25 years old. The samples and data may also form part of future research projects conducted by the researchers, but in this case, ethics approval with appropriate data custodian and institutional approvals will be sought before the samples or data are re-used. Study findings will be published in peer-reviewed journals and presented at national and international conferences.

**12. STATISTICAL CONSIDERATIONS AND DATA ANALYSIS**

**12.1 Sample size and statistical power**

Our project follows a pragmatic design to best allow the NPWT Pathway intervention to be tailored to the site context. The study will be conducted across four state’s tertiary paediatric burns centres. All sites will start the trial in control phase with the baseline measures taken, then one site will randomly be allocated (i.e., NPWT implemented across the site) (using computer-generated, centralised randomisation) to implementation every 6 weeks, until the new intervention has been implemented across all sites at 13 months. At 13 months after implementation has occurred across all sites, adherence sustainability will be measured to inform implementation effectiveness. Using a step wedge study design, and four sites with a minimum of 100 individuals recruited from each site (recruiting patients until the capacity of the site is reached), we would be able to detect a difference of 10% in adherence rate with 94% power (5% alpha). We expect an improvement of 10% in adherence from 10% in controls to 20% in the treatment. A greater difference in adherence, with the same parameters otherwise, will result in higher power and thus the above is the minimum numbers expected. Calculations were performed with the shiny app from Hemming et al. (2015) (38). We anticipate a minimum of 120 potential patients per site, which will be more than the above estimate and thus the power of this study is expected to be >94%.

**12.2 Statistical analysis methods**

Generalised Linear Mixed Models (fitting a random intercept to account for clustering, random effect) and Generalised Estimating Equations (using the individual as the clustering unit) will be used to compare the effect of switching from the usual care to INPREP condition on primary and secondary outcomes. Specifically, an appropriate protocol for model selection based on Zuur *et al*. (2009) will be applied (49). This analysis approach is able to account for potential temporal trends, using time as a covariate, in addition to clinical explanatory variables (total body surface area, age, gender), which will be included in each model. It should be noted that other covariates, such as time, could be used as random effects in the mixed models.

**12.2.1 Implementation evaluation**

We will use a previously described approach informed by the Consolidated Framework for Implementation Research (CFIR) (35). We will collate data from focus groups, questionnaires as well as field notes of clinicians, taken by the ReNs documenting feedback at the time of the intervention implementation. The transcribed interviews and field notes will be analysed using a deductive qualitative content analysis technique recommended for CFIR. The analysis will be performed by two researchers independently using the CFIR NVivo template pre-populated with construct codes – NVivo is a qualitative data analysis computer software package produced by QSR International [QSR International Pty Ltd. (2020) NVivo (released in March 2020) [https://www.qsrinternational.com/nvivo-qualitative-data-analysis-software/home](https://www.qsrinternational.com/nvivo-qualitative-data-analysis-software/home?_ga=2.227843295.371367470.1635126007-457000355.1635126007)]. The outputs of the analysis will be actionable recommendations to optimise the pathway and improve implementation.

**12.2.2 Cost effective analysis**

## We will record data, analyse, and report cost-effectiveness findings following guidelines for trial-based cost-effectiveness analyses (50), including reporting resource use and costs (healthcare perspective) for each trial condition. Healthcare utilisation data will be costed using actual costs (e.g., device costings) when available or market rates. Intervention provision costs to deliver usual care or INPREP during the trial (12-month time-horizon for patients) will be recorded and applied at a per-patient level. A trial-based incremental cost-effectiveness ratio (ICER) will be estimated for the incremental cost per additional patient successfully completing NPWT. ICER=[(CostNPWT) minus (Costusual care)] / [(EffectNPWT minus Effectusual care)]. Due to the potential for uncertainty and non-normal distributions, 95%CIs (for costs and effect estimates) and a 95% confidence ellipse (for ICER) will be derived from bootstrap resampling. In addition, Markov modelling (5-year time-horizon) will be used to extend these cost-effectiveness findings by generating estimates for the consequences (on costs and patient outcomes listed above) of implementing INPREP at all specialist paediatric hospitals in Australia using data from this trial and prior studies as well as health service and population profile data (e.g. Australian Bureau of Statistics). In addition to probabilistic sensitivity analyses, we will assess sensitivity of results to variation in measured resource use, unit costs, effectiveness, time-horizon, and discounting (one way and multi-way sensitivity analyses).

**13. ETHICAL CONSIDERATIONS**

Central ethical approval will be obtained from Children’s Health Queensland (CHQ) HREC via Ethics Review Manager with accompanying Victorian Specific Module (VSM) and Western Australian Specific Module (WASM). Expedited affiliated academic institution ethics approval will be sought from (Griffith University, University of NSW, Queensland University of Technology, University of Western Australia, and Monash Children’s Research Institute). A site-specific agreement (governance) will also be obtained for QCH. This is a national multi-centre investigation employing a hybrid type III effectiveness-implementation design. Four major Australian paediatric burn centres are participating:

- The Children’s Hospital at Westmead (CHW)
- Perth Children’s Hospital (PCH)
- Queensland Children’s Hospital (QCH)
- Royal Children’s Hospital Melbourne (RCHM)

The QCH is the lead site for this multi-centre investigation. Initial HREC approval will be sort by the lead site, and subsequently shared with the other three sites (The Children’s Hospital at Westmead, Royal Children’s Hospital Melbourne, and Perth Children’s Hospital). Each site will then be responsible for submitting local Governance and site-specific agreements. This study will be performed in accordance with the ethical principles of the Declaration of Helsinki, ICH GCP for Guidance on Good Clinical Practice and National Health and Medical Research Council (NHMRC) National Statement on Ethical Conduct in Research Involving Humans (NHMRC, Australian Research Council, and Australian Vice-Chancellors' Committee, 2007; World Medical Association, 2008) (51-53). No part of the study will commence prior to notifying CHQ HREC and governance.

**13.1 Safety considerations/Patient safety**

In accordance with ICH Good Clinical Practice guidelines and national regulations, all adverse events (including those not directly related to the child’s burn) will be recorded and submitted to the appropriate HREC and governing bodies. An adverse event (AE) is defined as any untoward, unfavourable, or unintended medical occurrence in a patient administered an investigational product – which does not necessarily have a causal relationship with the medicinal investigational product. Research and data collection methods outlined in this protocol have been included in previous studies at QCH with no adverse events. Although AE are not anticipated, Dr Alex De Young who is a practicing child psychologist with extensive experience with this population in this environment will be involved in the co-design process to minimise any potential distressing triggers for patients and/or their parents/carers. There will also be resources available for families to access throughout the study, including the burns social worker or referral to lifeline etc. The investigators at each partnering hospital will be responsible for recording all adverse events – regardless of their causal attribution to the burn NPWT intervention, with the following exceptions:

- Conditions and comorbidities that are present at initial screening following patient presentation and do not deteriorate will not be considered adverse events
- Abnormal laboratory values (from biospecimen samples) will not be considered adverse events unless deemed clinically significant by the site investigator

Investigators at each site will obtain information on AEs at each patient contact, and all AEs will be recorded in the patient’s medical records. Adverse Events Grade 2 or higher will be reported to the lead HREC and sponsor immediately, as per reporting requirements (see Table 1 below for AE grade definitions).

Table 1. Adverse Event Grading Scale

| **AE Grade** | **Severity** | **Unique Clinical Descriptors** |
| --- | --- | --- |
| Grade 1 | Mild | Asymptomatic or mild symptoms; clinical or diagnostic observations only; intervention not indicated |
| Grade 2 | Moderate | Minimal, local or non-invasive intervention indicated; limiting age-appropriate instrumental activities of daily living |
| Grade 3 | Severe or medically significant but not immediately life-threatening | Hospitalisation or prolongation of hospitalisation indicated; disabling; limiting self-care activities of daily living |
| Grade 4 | Life-threatening consequences | Urgent intervention indicated |
| Grade 5 | Death related to AE | Death |

*Adverse event terminology and descriptors presented above in Table 1 were taken from Version 5 of the *Common Terminology Criteria for Adverse Events,* US Department of Health and Human Services, National Cancer Institute (2017).

<https://ctep.cancer.gov/protocoldevelopment/electronic_applications/docs/ctcae_v5_quick_reference_8.5x11.pdf>

**13.2 Withdrawal from project**

Participants (patients, parents/caregivers, and clinicians) may withdraw from the study at any time for any reason. Patients, parents, and caregivers will be reassured that withdrawal will not affect their routine treatment or relationship with those treating them or relationship with participating sites. Clinician participants will be reassured that withdrawal will not affect their relationship with their employers or management team. Once the decision to withdraw has been made, participants will also be given the option to have their previously collected data removed. The reidentification for removal may occur locally and the central data collection team are provided only with the study number for the participant. We will ask the participants (clinician or patient) if they are comfortable with the investigators keeping their data collected to date for research purposes, or alternatively, if they are withdrawing participation prospectively and previously collected data

**14. DISSEMINATION**

Following data analysis and write-up, the study report will be submitted to a peer-reviewed medical journal, preferably under an open access format to permit free access to the published study. During surveys and focus groups, participants can elect to receive a newsletter and or a project completion report, summarising the findings. De-identified raw data will be stored within a data repository and made available to other researchers upon reasonable request and following review of such a request by the original ethics committees. Results from this investigation will also be presented at local and international medical conferences and disseminated to relevant stakeholders and knowledge users at each of the Australian sites.

**15. SIGNIFICANCE**

Not only will this study achieve the goals of the clinical partner (such as embedding evidence-based care and improving patient outcomes), but this study will also inform policy and practice nationally on how NPWT for children with acute burns can be implemented into various contexts of burn care. Whilst significant morbidity still exists for children with burns, innovative, cost effective, and sustainable solutions are required to decrease healing time and decrease the risk of scarring. This funding application provides a practical solution to a national problem. Evidence generated will inform paediatric burn care internationally. Through rigorous implementation methodology, this project will impact and sustain the INPREP Pathway beyond the funded study. This project will be the world’s first large-scale study to evaluate the implementation of NPWT for paediatric burns. Other key project outcomes will include:

- 1. Map of Australian acute paediatric burn care models of care and co-designed INPREP Pathway to nest within it
  2. Evidence-based implementation strategies
  3. Improved patient outcomes
  4. Identification of the biological and biochemical mechanisms underpinning the effectiveness of NPWT on acute burn wounds
  5. Development and distribution of education/training resources
  6. Cost-effective implementation
  7. Standardisation (sensitive to context)
  8. Comprehensive dissemination using innovative and diverse approaches via our website, training packages, social media/podcasts, webinars to implement NPWT and achieve benefits broadly and sustainably

**15. REFERENCES**

1. Peck MD. Epidemiology of burns throughout the world. Part I: Distribution and risk factors. Burns. 2011;37(7):1087-100.

2. Deitch EA, Wheelahan TM, Rose MP, Clothier J, Cotter J. Hypertrophic Burn Scars: Analysis of Variables. The journal of trauma. 1983;23(10):895-8.

3. Lonie S, Baker P, Teixeira RP. Healing time and incidence of hypertrophic scarring in paediatric scalds. Burns. 2016;43(3):509-13.

4. Chipp E, Charles L, Thomas C, Whiting K, Moiemen N, Wilson Y. A prospective study of time to healing and hypertrophic scarring in paediatric burns: Every day counts. Burns and trauma. 2017;5(1):3-.

5. Wallace HJ, Fear MW, Crowe MM, Martin LJ, Wood FM. Identification of factors predicting scar outcome after burn injury in children: A prospective case-control study. Burns and trauma. 2017;5(1):19-.

6. Dedovic Z, Koupilová I, Brychta P. Time trends in incidence of hypertrophic scarring in children treated for burns. Acta chirurgiae plasticae. 1999;41(3):87-90.

7. Cubison TCS, Pape SA, Parkhouse N. Evidence for the link between healing time and the development of hypertrophic scars (HTS) in paediatric burns due to scald injury. Burns. 2006;32(8):992-9.

8. Bartlett N, Yuan J, Holland AJA, Harvey JG, Martin HCO, La Hei ER, et al. Optimal duration of cooling for an acute scald contact burn injury in a porcine model. Journal of burn care & research. 2008;29(5):828-34.

9. Cuttle L, Kempf M, Liu P-Y, Kravchuk O, Kimble RM. The optimal duration and delay of first aid treatment for deep partial thickness burn injuries. Burns. 2009;36(5):673-9.

10. Gee Kee EL, Kimble RM, Cuttle L, Khan A, Stockton KA. Randomized controlled trial of three burns dressings for partial thickness burns in children. Burns. 2014;41(5):946-55.

11. Griffin BR, Frear CC, Babl F, Oakley E, Kimble RM. Cool Running Water First Aid Decreases Skin Grafting Requirements in Pediatric Burns: A Cohort Study of Two Thousand Four Hundred Ninety-five Children. Annals of emergency medicine. 2019;75(1):75-85.

12. Wood FM, Phillips M, Jovic T, Cassidy JT, Cameron P, Edgar DW. Water First Aid Is Beneficial In Humans Post-Burn: Evidence from a Bi-National Cohort Study. PloS one. 2016;11(1):e0147259.

13. Frear CC, Griffin BR, Cuttle L, Kimble RM, McPhail SM. Cost-effectiveness of adjunctive negative pressure wound therapy in paediatric burn care: evidence from the SONATA in C randomised controlled trial. Scientific reports. 2021;11(1):16650-.

14. Frear CC, Griffin B, Cuttle L, McPhail SM, Kimble R. Study of negative pressure wound therapy as an adjunct treatment for acute burns in children (SONATA in C): Protocol for a randomised controlled trial. Trials. 2019;20(1):130-.

15. Kamolz LP, Andel H, Haslik W, Winter W, Meissl G, Frey M. Use of subatmospheric pressure therapy to prevent burn wound progression in human: first experiences. Burns. 2004;30(3):253-8.

16. Morykwas MJ, David LR, Scneider AM, Whang C, Jennings DA, Canty C, et al. Use of subatmospheric pressure to prevent progression of partial- thickness burns in a swine model. Journal of burn care & rehabilitation. 1999;20(1 I):15-21.

17. Schrank C, Mayr M, Overesch M, Molnar J, Henkel VDG, Mühlbauer W, et al. [Results of vacuum therapy (v.a.C.) of superficial and deep dermal burns]. Zentralblatt fur Chirurgie. 2004;129 Suppl 1:S59-61.

18. World Health Organisation. Global Burden of Disease Geneva2008.

19. Burns Registry of Australia and New Zealand (2021). Annual Report 2019/20. Department of Epidemiology and Preventive Medicine, Monash University. Melbourne, Australia.

20. Greenhalgh DG. Management of Burns. The New England journal of medicine. 2019;380(24):2349-59.

21. Wang Y, Beekman J, Hew J, Jackson S, Issler-Fisher AC, Parungao R, et al. Burn injury: Challenges and advances in burn wound healing, infection, pain and scarring. Advanced Drug Delivery Reviews. 2018;123:3-17.

22. Nunez Lopez O, Cambiaso-Daniel J, Branski LK, Norbury WB, Herndon DN. Predicting and managing sepsis in burn patients: Current perspectives. Ther Clin Risk Manag. 2017;13:1107-17.

23. Guo S, DiPietro LA. Factors Affecting Wound Healing. J Dent Res. 2010;89(3):219-29.

24. Hop MJ, Polinder S, van der Vlies CH, Middelkoop E, van Baar ME. Costs of burn care: A systematic review. Wound repair and regeneration. 2014;22(4):436-50.

25. Zheng XP, Chen J, Chen TS, Jiang YN, Shen T, Xiao SC, et al. [Preliminary effect observation on the application of micro-negative pressure in children with small-area deep partial-thickness burn]. Zhonghua Shao Shang Za Zhi. 2019;35(10):720-5.

26. Frear CC, Cuttle L, McPhail SM, Chatfield MD, Kimble RM, Griffin BR. Randomized clinical trial of negative pressure wound therapy as an adjunctive treatment for small-area thermal burns in children. The British journal of surgery. 2020;107(13):1741-50.

27. Chen J, Zhou JJ, Su GL, Shi JW, Su SJ. [Evaluation of the clinical curative effect of applying vacuum sealing drainage therapy in treating deep partial-thickness burn wound at the initial stage]. Zhonghua shao shang za zhi = Zhonghua shaoshang zazhi = Chinese journal of burns. 2010;26(3):170-4.

28. Haslik W, Kamolz LP, Andel H, Meissl G, Frey M. [The use of subatmospheric pressure to prevent burn wound progression: first experiences in burn wound treatment]. Zentralbl Chir. 2004;129 Suppl 1:S62-3.

29. Molnar JA, Heimbach DM, Tredget EE, Hickerson WL, Still JM, Luterman A. Prospective randomized controlled multicenter trial applying subatmospheric pressure to acute hand burns: an interim report. 2004.

30. Damschroder LJ, Aron DC, Keith RE, Kirsh SR, Alexander JA, Lowery JC. Fostering implementation of health services research findings into practice: A consolidated framework for advancing implementation science. Implementation science : IS. 2009;4(1):50-.

31. Frear CC, Zang T, Griffin BR, McPhail SM, Parker TJ, Kimble RM, et al. The modulation of the burn wound environment by negative pressure wound therapy: Insights from the proteome. Wound repair and regeneration. 2021;29(2):288-97.

32. Powell BJ, Waltz TJ, Chinman MJ, Damschroder LJ, Smith JL, Matthieu MM, et al. A refined compilation of implementation strategies: Results from the Expert Recommendations for Implementing Change (ERIC) project. Implementation science : IS. 2015;10(1):21-.

33. Abella BS, Rhee JW, Huang K-N, Vanden Hoek TL, Becker LB. Induced hypothermia is underused after resuscitation from cardiac arrest: a current practice survey. Resuscitation. 2005;64(2):181-6.

34. Sasson C, Forman J, Krass D, Macy M, Kellermann AL, McNally BF. A qualitative study to identify barriers to local implementation of prehospital termination of resuscitation protocols. Circulation Cardiovascular quality and outcomes. 2009;2(4):361-8.

35. Keith RE, Crosson JC, O'Malley AS, Cromp D, Taylor EF. Using the Consolidated Framework for Implementation Research (CFIR) to produce actionable findings: a rapid-cycle evaluation approach to improving implementation. Implement Sci. 2017;12(1):15.

36. Waltz TJ, Powell BJ, Fernández ME, Abadie B, Damschroder LJ. Choosing implementation strategies to address contextual barriers: Diversity in recommendations and future directions. Implementation science : IS. 2019;14(1):42-.

37. Curran GM, Bauer M, Mittman B, Pyne JM, Stetler C. Effectiveness-implementation Hybrid Designs: Combining Elements of Clinical Effectiveness and Implementation Research to Enhance Public Health Impact. Medical care. 2012;50(3):217-26.

38. Hemming K, Haines TP, Chilton PJ, Girling AJ, Lilford RJ. The stepped wedge cluster randomised trial: rationale, design, analysis, and reporting. BMJ : British Medical Journal. 2015;350:h391.

39. Weiner BJ, Lewis CC, Stanick C, Powell BJ, Dorsey CN, Clary AS, et al. Psychometric assessment of three newly developed implementation outcome measures. Implementation Science. 2017;12(1):108.

40. ANZBA. Care [Available from: <https://anzba.org.au/care/first-aid/>]. 2020.

41. Holbert MD, Griffin BR, McPhail SM, Ware RS, Foster K, Bertoni DC, et al. Effectiveness of a hydrogel dressing as an analgesic adjunct to first aid for the treatment of acute paediatric thermal burn injuries: study protocol for a randomised controlled trial. Trials. 2019;20(1):13-.

42. Zang T, Cuttle L, Broszczak DA, Broadbent JA, Tanzer C, Parker TJ. Characterization of the Blister Fluid Proteome for Pediatric Burn Classification. Journal of proteome research. 2019;18(1):69-85.

43. Frear CC, Cuttle L, McPhail SM, Chatfield MD, Kimble RM, Griffin BR. Randomized clinical trial of negative pressure wound therapy as an adjunctive treatment for small‐area thermal burns in children. British journal of surgery. 2020;107(13):1741-50.

44. Mikhal'Chik EV, Piterskaya JA, Budkevich LY, Pen'Kov LY, Facchiano A, De Luca C, et al. Comparative study of cytokine content in the plasma and wound exudate from children with severe burns. Bulletin of experimental biology and medicine. 2009;148(5):771-5.

45. Moseley R, Hilton JR, Waddington RJ, Harding KG, Stephens P, Thomas DW. Comparison of oxidative stress biomarker profiles between acute and chronic wound environments. Wound repair and regeneration. 2004;12(4):419-29.

46. Zakaria R, Allen KJ, Koplin JJ, Roche P, Greaves RF. Advantages and Challenges of Dried Blood Spot Analysis by Mass Spectrometry Across the Total Testing Process. EJIFCC. 2016;27(4):288-317.

47. Zang T, Cuttle L, Broszczak DA, Broadbent JA, Tanzer C, Parker TJ. Characterization of the Blister Fluid Proteome for Pediatric Burn Classification. Journal of proteome research. 2019;18(1):69-85.

48. Parker TJ, Sampson DL, Broszczak D, Chng YL, Carter SL, Leavesley DI, et al. A Fragment of the LG3 Peptide of Endorepellin Is Present in the Urine of Physically Active Mining Workers: A Potential Marker of Physical Activity. PLOS ONE. 2012;7(3):e33714.

49. Zuur AF. Mixed effects models and extensions in ecology with R. 1. Aufl. ed. London;New York;: Springer; 2009.

50. Ramsey SDMDP, Willke RJP, Glick HP, Reed SDPR, Augustovski FMDMP, Jonsson BP, et al. Cost-Effectiveness Analysis Alongside Clinical Trials II—An ISPOR Good Research Practices Task Force Report. Value in health. 2015;18(2):161-72.

51. World Medical Association Declaration of Helsinki. 1964.

52. Note for guidance on good clinical practice (CPMP/ICH/135/95 - Annotated with TGA comments).

53. National Statement on Ethical Conduct in Human Research. 2007.

**16. APPENDICES**

**Appendix 1. Wound Fluid Collection, Processing, and Storage**

Method taken from A/Prof Cuttle’s Protocol: SOP-Wound-Fluid-v3-20200212

Purpose

This SOP describes the methodology for the collection and processing of burn wound fluid (WF) samples. Wound fluid samples are opportunistically collected from burn patients who present to the burn centre or emergency department with a burn injury. As part of routine clinical wound cleaning procedures, blisters are de-roofed, and blister fluid is removed and discarded. For this project, instead of being discarded, the blister fluid will be collected, processed and stored so that it can be analysed and used for burn wound healing studies. Additionally, if patients present with an exudating wound, this fluid will be collected instead of discarded. And if patients present with a negative pressure device attached to their wound, and there is fluid in the suction tube, this will also be collected. Samples are ideally received in volumes >100uL, although occasionally less is collected, as sample volumes as small as 50µL can still be analysed using mass spectrometry.

Aim

To process WF specimens and freeze in smaller aliquots to maintain protein quality and prevent repeated free-thawing of samples.

Reagents and Consumables

- Specimen pot 70mL, Sarstedt Cat#75.9922.745 (Polypropylene)
- Whatman Filter paper, sterilised
- Eppendorf Protein Lo-bind tubes Cat#30108094
- Filtered pipette tips
- Pipettes – P200 and P1000
- Microcentrifuge, for 1.5mL to 2.0mL tubes
- Site-specific participant code book or REDCap database
- De-identified wound fluid sample logbook or REDCap database
- Brady label maker Cat#BMP51

Standard Personal Protective Equipment (PPE) must be worn at all times when collecting and processing biological specimens, including gloves and safety glasses during collection and gloves, safety glasses and a laboratory gown when processing the samples in the laboratory. Any staff member or student who is involved in the processing of biological samples must be immunised against Hepatitis B.

Procedure

- 1. During routine wound cleaning procedures, the clinical/nursing team will lance patient blisters and remove any exudating wound fluid. If there is a large volume of fluid (e.g. several mLs,), the fluid can initially be collected in a larger container, such as a specimen pot temporarily.

If the samples are smaller volume, they can be collected directly into the Eppendorf Protein Lo-bind tubes.

If there are multiple blisters on the same patient, these should be collected in separate tubes/pots.

If the patient has a negative pressure device with fluid in the tubing, the tube can be clamped at either end, cut off the device and the fluid from the tube collected in a specimen pot.

If there is only a small amount of fluid, this can be collected with filter paper. The filter paper is placed at the wound edge to absorb the fluid, and then placed into sterile 2mL Lo-Bind tubes.

If a sample is collected in a specimen pot, please transfer to a Lo-bind tube ASAP to prevent protein loss.

- 1. Label the tube/pot with patient UR sticker, the anatomical location of the blister or wound site (e.g. right palm, left thigh) and collection date. **Store in a fridge at 4°C if not processed immediately, and process within 24 hours.**
  2. If the sample is in a specimen pot, transfer it into Eppendorf Protein Lo-bind tube/s ASAP. If you transfer it into multiple tubes (i.e. the volume is greater than 2.0 mL), transfer equal amounts into each tube, so that they will balance in the centrifuge. Label the tube with the participant unique identifier code from your site-specific participant code book or from the REDCap database.

e.g. 49_ 23_ WFa_20190408

(Brisbane Code_ Participant #23_ Wound Fluid__Anatomical Area abcde_Date yyyymmdd)

If the sample is in filter paper, recover the wound fluid by eluting the sample in 100µL of Phosphate Buffered Saline or water at 4◦C for 1 hour (45).

- 1. Centrifuge the sample in a balanced benchtop microfuge for 3 minutes at 855 x x g at ambient temperature to pellet cells and cellular debris.
  2. Record the collection date, anatomical location, total sample volume and any comments on the fluid contents (Blood? Colour? Consistency?) in the WF sample logbook or in the REDCap database.
  3. Without disturbing the pellet, transfer the supernatant to fresh Lo-bind tubes. The volume in each tube should be minimum 50µL, to a maximum of 1000µL. Ensure there are at least 2 aliquots (2 tubes) of each sample. Write the total number of aliquots and aliquot volumes in the WF sample logbook or REDCap database.
  4. Label each aliquot with sample number and date, using the Brady label maker.
  5. Store aliquots in a -80°C freezer.

**Appendix 2. Blood Collection, Processing, and Storage**

Method taken from A/Prof Cuttle’s Protocol: SOP-Blood-Processing-5-2020200421

Purpose

This SOP describes the methodology for the collection and processing of blood samples. Blood samples are opportunistically collected from burn patients who are undergoing a general anaesthetic for routine burn injury treatments (e.g. dressing change, debridement or grafting). The volume of blood that can be collected from a child depends on their age/size and total blood volume. According to the WHO guidelines for the safe limits of blood volume collection from children, 1-5% of total blood volume can be collected within a 24-hour period, with a maximum of 3mL per kg for sick children. To obtain enough cells to process, collect a minimum of 1mL blood volume, ideally collect around 8-10mL.

Aim

To collect and process blood specimens to obtain Peripheral Blood Mononuclear Cells (PBMC) and plasma. These samples will then be frozen for later analysis.

Reagents and Consumables

- Vacutainers containing lithium heparin (e.g. Becton Dickinson green 10mL Cat #367874)
- Eppendorf Protein Lo-bind tubes Cat#30108094
- Lymphoprep or Ficoll-paque Plus – brought to room temperature
- RPMI containing 20% Heat-inactivated fetal calf serum FCS/FBS – brought to room temperature
- 15mL and 50mL polypropylene tubes
- Sterile, filtered pipette tips
- Pipettes – P200 and P1000
- Electronic pipette aid and serological pipettes (2mL – 25mL)
- Vacuum suction and glass pipettes
- DMSO
- Cryovials, 2ml Corning CLS430659, these cryovials are for LN2 Vapour storage.
- CoolCell/MrFrosty
- Site-specific participant code book or REDCap database
- De-identified blood sample logbook or REDCAP database
- Brady label maker Cat#BMP51

Standard Personal Protective Equipment (PPE) must be worn at all times when collecting and processing biological specimens, including gloves and safety glasses during collection and gloves, safety glasses and a laboratory gown when processing the samples in the laboratory. Any staff member or student who is involved in the processing of biological samples must be immunised against Hepatitis B.

Procedure – Plasma and PBMC

1. Collect blood into vacutainers containing lithium heparin (the BD vacutainers we use specify “17 international units of heparin/mL of blood”). Aim to collect between 8-10mL of blood. Label tube with participant unique identifier code from your site-specific participant code book or REDCap database, and collection date and time.

**Process immediately if possible. Store at room temperature for up to an hour, for longer storage than an hour place in the fridge, and process within 24 hours.**

1. Record the collection and processing date and time in your site-specific participant code book or REDCap database. Centrifuge blood at 400 x g for 10min, brake ON. (400G=1340RPM, where radius=20cm)
2. Aliquot the plasma supernatant into 3-4 x Eppendorf Lo-bind tubes and label these with the unique patient code from your site-specific patient code book or REDCap database, using the Brady label maker.

e.g. 49_23_ P_20190408

(Brisbane Code_Participant #23_Plasma_Date yyyymmdd)

Record the collection date and total plasma volume in the blood sample log book or RedCap database. Store plasma aliquots in a -80 freezer.

Vacutainer Plasma and Blood fractions:


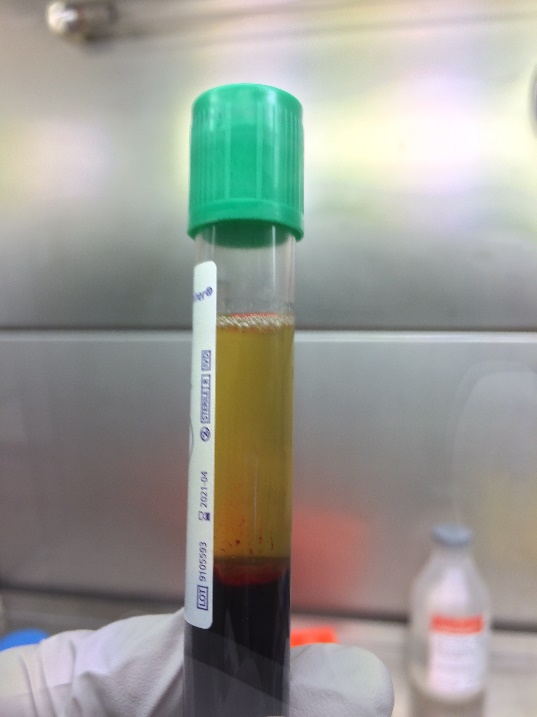


Plasma fraction for collection. Do not disturb interphase with RBCs, leave some plasma behind in tube

1. Dilute the remaining blood cells up to 4x in room temperature RPMI+20%FCS. It will probably be necessary to make a 2x dilution first, transfer to a 10 or 15mL tube, then dilute 2x again. * Dilute the remaining cells by first replacing the volume of plasma you removed, i.e. If you removed 4ml of plasma – place 4mls of RPMI & FBS into the remaining blood sample. Secondly, dilute the whole sample 1:1 with RPMI&FBS.
2. Prepare 2 x 15mL tubes by pipetting the correct volume of room temperature Ficoll-paque Plus (or Lymphoprep). See below for volumes (note: use regular 15ml tubes in place of Sepmate tubes):


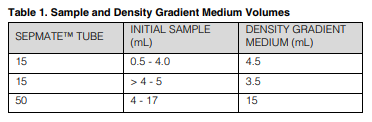


1. Split the diluted blood equally between the two tubes, carefully pipetting down the sides of the tubes.

Blood & Lymphoprep fractions pre-centrifugation


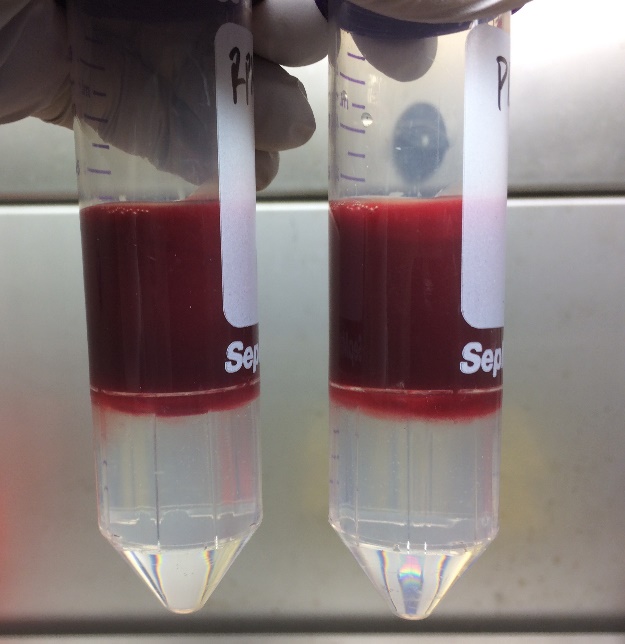


1. Centrifuge the sample at 400 x g for 30min at room temperature with the **brake OFF**. (400G=1340RPM, where radius=20cm)

Tubes post spin should look similar to these;


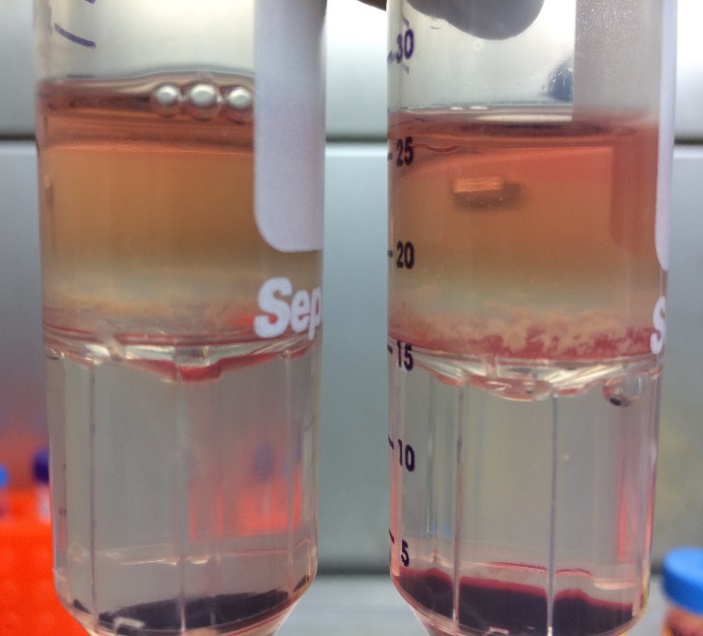

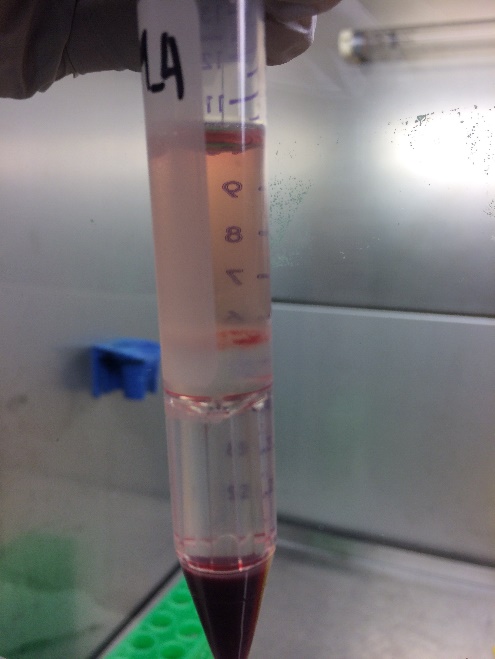


After centrifugation

Clear PBMC fraction to collect

1. For isolation of the buffy coat layer (containing PBMCs), carefully insert your pipette down the side of the tube and remove out the buffy coat layer. Resuspend into 2 x 15mL tubes.
2. Make each tube up to 15mL with RPMI + 20% FCS. Centrifuge at 500 x g for 10min, brake on. (500G=1500RPM, where radius=20cm).

PBMCs should be a pellet like this:


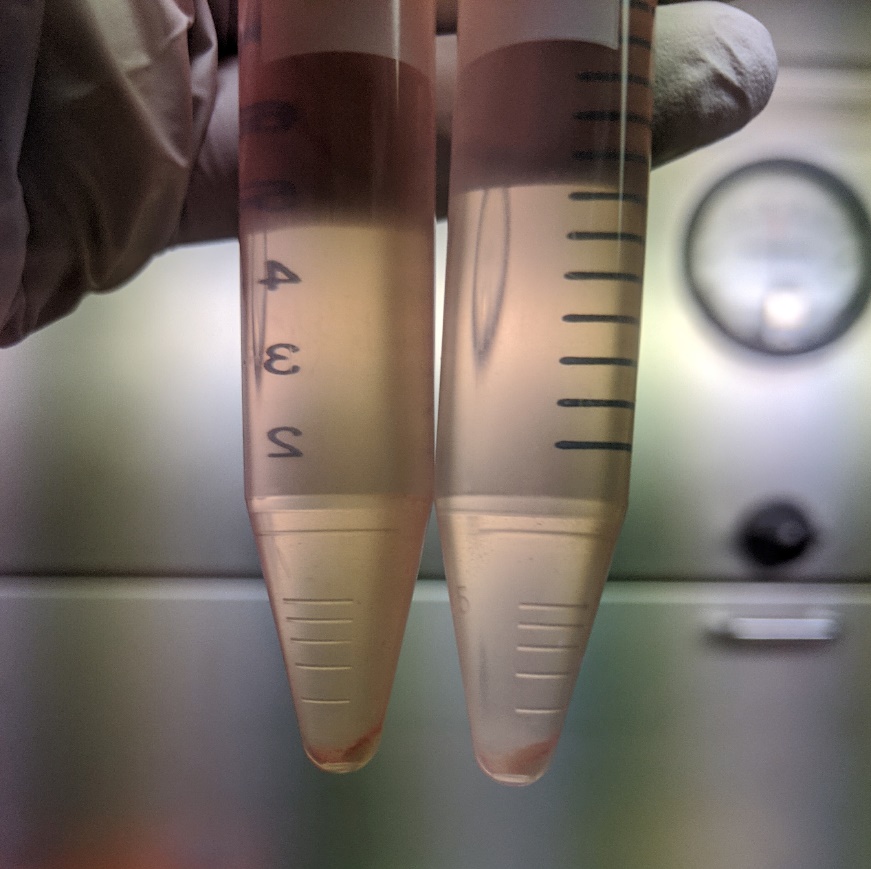


1. Discard the supernatants. Resuspend the pellet from one tube in 1mL of RPMI + 20% FCS and transfer to the other tube. Resuspend the cells in 15mL of RPMI + 20% FCS and centrifuge at 120 x g for 10min, **brake OFF.** This will deplete the platelets from the samples.
2. Discard the supernatant and resuspend the cells in 1mL RPMI + 20% FCS. Take an aliquot for counting.
3. The method used to count the cells is not important as long as it is accurate and allows for live/dead discrimination. We will count by taking a 1:10 dilution of cells, further diluting them 1:2 in 0.4% trypan blue, then counting manually using a haemocytometer.
4. Cells will be stored at approximately 2 x 10^6^ cells/vial. Using the label maker, label enough cryovials with the participant identifier code and place them on ice.

e.g. 49_ 23_ C_20190408

(Brisbane Code_Participant #23_Cells_Date yyyymmdd)

1. Dilute the cells with RPMI + 20% FCS to a concentration of approximately 2 x 10^6^ cells/mL per vial.
2. Place the cells on ice and add an equal volume of pre-chilled RPMI + 15% DMSO; take at least 1min to add the first mL of 15% DMSO drop-by-drop, and slowly add the rest.
3. Transfer aliquots to the labelled cryovials.
4. Move cryovials into a CoolCell/MrFrosty (at room temp) and store in a -80C freezer, at least overnight.
5. Transfer cryovials to liquid nitrogen storage. Write details of the number of cryovials in the blood sample logbook or REDCap Database.

**Appendix 3. Hair Collection, Processing and Storage**

Method based on Treya Long Protocol 2020

Collecting Hair Samples

1. Separate the hair at the back of the head with a hair clip
2. Collect a hair strand of about 3-5mm and comb
3. Tie up the hair strand with a small hair band/tie or string close to the scalp
4. Cut the hair as close as possible to the scalp
5. Place the hair into the 2ml screw-cap cryo tube

Record Keeping

Record the following in lab book:

1. Patient number
2. Date of sample processing
3. Time of sample processing
4. Name of scientist processing

Hair Storage

Tube should be labelled as follows:

- Patient Number_Sample Date (ddmmyyyy)_H

For example: 1_26032020_H)

- Hair should be stored directly at -80°C

**Appendix 4. Urine Collection, Processing and Storage**

Purpose

This SOP describes the methodology for the collection and processing of urine samples. Urine samples are opportunistically collected from burn patients who present to the burn centre or emergency department with a burn injury. If participants are scheduled to receive a urinary catheter as part of routine standard treatment for their burns, some urine that will be retained for analysis. For participants who do not require a catheter, the child and parent/caregiver will be asked if they consent to providing a urine sample and will be given a urine sample pot container for collection. A minimum volume of 60mL is required for analysis.

Aim

To process urine specimens and freeze in smaller aliquots to maintain protein quality and prevent repeated freeze-thawing of samples.

Reagents and Consumables

- Sterile Specimen pot 250mL, Sarstedt Cat#75.9922.745 (Polypropylene)
- 3 kDa NMWL Amicon® ultra-15 centrifugal filter devices
- Eppendorf Protein Lo-bind tubes Cat#30108094
- Filtered pipette tips
- Pipettes – P200 and P1000
- Microcentrifuge, for 1.5mL to 2.0mL tubes and 50mL tubes
- Site-specific participant code book or REDCap database
- De-identified urine sample logbook or REDCap database
- Brady label maker Cat#BMP51

Standard Personal Protective Equipment (PPE) must be worn at all times when collecting and processing biological specimens, including gloves and safety glasses during collection and gloves, safety glasses and a laboratory gown when processing the samples in the laboratory. Any staff member or student who is involved in the processing of biological samples must be immunised against Hepatitis B.

Procedure

1. A mid-stream urine sample (if possible) will initially be collected in a sterile specimen pot.
2. Label the tube with the participant unique identifier code from your site-specific participant code book or from the REDCap database.

e.g. 49_ 23_ U_20190408

(Brisbane Code_ Participant #23_ Urine_Date yyyymmdd)

1. Freeze the sample at -20^◦^C until processing the next day or later.
2. Thaw the sample in a 25^◦^C water bath, and transfer the sample into multiple 50mL Falcon tubes.
3. Centrifuge the tubes at 1,500 × g for 10 minutes. Collect the supernatant and pass through a sterile 0.2 μm syringe filter to remove any remaining bacterial contamination and high molecular weight protein aggregates.
4. Collect filtrate and concentrate by centrifugation in a pre-rinsed 3 kDa NMWL Amicon® ultra-15 centrifugal filter device, as per manufacturer’s instructions, at 4,000 × g for 30 minutes.
5. Dilute the retentate with 15 mL milli-Q water and centrifuge twice at 4,000 × g for 30 minutes to desalt and remove low molecular weight non-protein components.
6. Following sample concentration, transfer 1mL aliquots of the retentate of each sample into Lo-bind tubes. Label each aliquot with sample number and date, using the Brady label maker.
7. Store aliquots in a -80°C freezer.
8. Write the total number of urine aliquots and aliquot volumes in the sample logbook or REDCap database.

**Appendix 5. Schedule of Assessments**

| Visit | Initial Presentation | COD1 | COD2, COD3…^1^ | Surgical intervention | 3 months | 12 months |
| --- | --- | --- | --- | --- | --- | --- |
| Eligibility and consent |  |  |  |  |  |  |
| Eligibility screen | X |  |  |  |  |  |
| Signed informed consent | X |  |  |  |  |  |
| REDCap data collection forms |  |  |  |  |  |  |
| Demographic and Injury Details | X |  |  |  |  |  |
| Initial Presentation | X |  |  |  |  |  |
| First Change of Dressing |  | X |  |  |  |  |
| Ongoing Dressing Changes |  |  | X |  |  |  |
| Surgical Interventions & Requirements |  |  |  | X |  |  |
| Completion Data |  |  |  |  |  | X |
| Additional data points |  |  |  |  |  |  |
| Time taken to complete dressing | X | X |  |  |  |  |
| % Re-epithelialised | X | X | X | X |  |  |
| Burn depth re-assessment |  | X |  |  |  |  |
| Photos^2^ | X | X | X | X |  |  |
| Pain scales |  |  |  |  |  |  |
| Clinician pain scales – FLACC/NRS^3^ |  |  |  |  |  |  |
| Baseline | X | X | X | X |  |  |
| Peak procedural | X | X | X |  |  |  |
| Parent-proxy pain scales - NRS |  |  |  |  |  |  |
| Baseline | X | X | X | X |  |  |
| Peak procedural | X | X | X |  |  |  |
| Patient pain scales – FACES^4^ |  |  |  |  |  |  |
| Baseline | X | X | X | X |  |  |
| Peak procedural | X | X | X |  |  |  |
| Implementation outcomes^5^ |  |  |  |  |  |  |
| Clinician | X | X |  |  |  |  |
| Parent-Guardian |  | X |  |  |  |  |
| Biological Samples |  |  |  |  |  |  |
| Urine Sample | X | X |  |  |  |  |
| Blood Sample^6^ | X | X | X | X |  |  |
| Wound Fluid | X | X^7^ |  |  |  |  |
| Hair Sample | X |  |  |  | X |  |

COD = change of dressing; FLACC = face, legs, activity, cry, consolability; NRS = numeric rating scale; FACES = faces pain scale – revised

^1^ Every change of dressing until patient is 95% re-epithelialised.

^2^ Wound photos to be taken after debridement but before dressing application.

^3^ FLACC to be collected if patient under 7 years of age, NRS to be collected if patient ages 7 or over.

^4^ Patient pain scales only required for patients aged 7 years or older.

^5^ Implementation outcomes only required for patients enrolled in the intervention arm.

^6^ Only if patient is already cannulated or undergoing procedure under a general anaesthetic.

^7^ Only if patient in intervention arm and wound fluid able to be collected from NPWT device tubing.
