# Supplementary material for: Implementation of negative pressure for acute pediatric burns (INPREP): A stepped-wedge cluster randomized controlled trial protocol
Source: PLoS One. 2024 Dec 10;19(12):e0315278. doi: 10.1371/journal.pone.0315278 (PMC11630585; doi:10.1371/journal.pone.0315278)
Supplement: S1 File — (DOCX) [file pone.0315278.s002.docx]

**Negative Pressure Wound Therapy (NPWT) Decision Pathway**

**Revert to standard care** Document in notes why patient not suitable for NPWT under INPREP Pathway

**Not suitable for NPWT application** under the INPREP Pathway

**Is this an acute burn injury?** E.g. ≤48 hours since time of injury

**NO**

1. **Acute Burn Injury**

**YES**

**INFORMATION BOX**

**Anatomical Considerations:**

- Potential anatomical exclusions/considerations (i.e., eyes, face, genitalia, mucosal surfaces, circumferential neck)

**Contextual Considerations:**

- Medical considerations (i.e., infections, vascular conditions, coagulopathy​, allergy to adhesive fixation​)
- Psychosocial considerations (i.e., pre-existing diagnoses, complex social situations, unable to take care of device, geographical location)

*[Your site may have additional anatomical exclusions - please check and modify]*

**YES**

**YES**

**Revert to standard care** Document in notes why patient not suitable for NPWT under INPREP Pathway

**NO**

**NO**

**NO**

**YES**

Consider appropriateness of NPWT under the INPREP Pathway

Refer to the after-hours information file for your specific site

**Is it after-hours?** E.g., Outside of 7.30am – 4pm

**Are there any contextual considerations that may make the patient unsuitable?** *Refer to information box*

Consider appropriateness of NPWT under the INPREP Pathway

Please consent and enrol patient in trial or contact the Research Officer on: 04XX XXX XXX

Patient suitable for NPWT under INPREP Pathway

1. **Anatomical** **Regions**
2. **Contextual Considerations**

**Is the anatomical region suitable?** *Refer to information box*
