# Supplementary material for: Implementation of negative pressure for acute pediatric burns (INPREP): A stepped-wedge cluster randomized controlled trial protocol
Source: PLoS One. 2024 Dec 10;19(12):e0315278. doi: 10.1371/journal.pone.0315278 (PMC11630585; doi:10.1371/journal.pone.0315278)
Supplement: S2 File — (DOCX) [file pone.0315278.s003.docx]

**COMMENCEMENT AND MANAGEMENT OF NEGATIVE PRESSURE WOUND THERAPY (NPWT) UNDER THE INPREP TRIAL PATHWAY**

1. **PURPOSE**

This document outlines the procedural requirements for the application of a Negative Pressure Wound Therapy (NPWT) device in the management of acute paediatric burn injuries, under the INPREP Trial Pathway.

1. **SCOPE AND TARGET AUDIENCE**

This procedure applies to all Medical and Nursing staff within [INSERT Hospital and Health Service] who will be applying NPWT to children with acute burns as part of the INPREP Trial Pathway.

1. **BACKGROUND**

Negative Pressure Wound Therapy (NPWT), also known as vacuum-assisted closure, is a therapeutic technique used in the management of acute and chronic wounds. It involves the application of controlled subatmospheric pressure to a wound to enhance wound healing and promote tissue regeneration. NPWT has been increasingly utilised in the treatment of various types of wounds, including acute burns, to optimise wound healing outcomes. This controlled negative pressure helps to:

- Remove excess fluid and inflammatory mediators from the wound, reducing the risk of infection and optimising the wound environment for healing
- Promote tissue granulation
- Reduce swelling, improving nutritional and oxygen delivery to the wound and promoting tissue perfusion
- Bring the wound edges closer together, reducing dead space and promoting wound closure

NPWT has shown to accelerate burn wound re-epithelialisation by up to 2-days^1^. In the context of acute burns, NPWT can be beneficial during the early stages of burn management, specifically in cases where there is a need for excision and grafting procedures.

1. **CONTEXTUAL CONSIDERATIONS**

Consider the following patient factors and contextual considerations that may exclude a child from receiving NPWT under the INPREP Trial Pathway. These **ARE NOT** definitive exclusions – please consider the following factors and use your clinical judgement to determine the appropriateness of enrolling the patient in the INPREP trial:

| **MEDICAL**   - Infections​ - Vascular conditions ​ - Coagulopathy​ - Known allergy to adhesive fixation​ - Deep burns with exposed tendons, structures, and/or blood vessels​ - Circumferential deep-dermal or full-thickness burns excluded at the discretion of treating clinician to assess capillary refill ​ - Erythema only burns​ - Patient experiencing significant pain post-burn |
| --- |
| **PSYCHOSOCIAL**   - State residential care​ - Non-English-speaking background without access to interpreter for informed consent​ - Responsibility to take care of device (parents, caregivers, and patients)​ - Pre-existing diagnoses (behavioural, cognitive, or developmental issues/difficulties affecting compliance)​ - Complex social situations (i.e., mental health, chaotic family dynamic, unstable home environment)​ - Geographical considerations (i.e., regional, rural, and remote families with limited local support) |
| **ANATOMICAL**   - Eyes​ - Face​ - Genitalia​ - Mucosal surfaces (i.e., anus, lips, stoma)​ - Circumferential neck |

1. **TYPES OF NPWT PUMPS AVAILABLE WITHIN [INSERT LOCAL HHS]**

***Remove NPWT Devices not available within your local HHS***

| **CANISTER DEVICES** | Description |
| --- | --- |
| 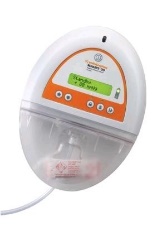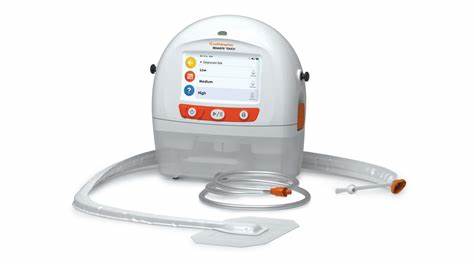RENASYS GO/TOUCH (Smith & Nephew) | - The device-battery lasts approximately 20-hours. - The device has both audible and visual alarms. - If the absorbent pillow inside the canister ruptures, this is not a concern. |
| 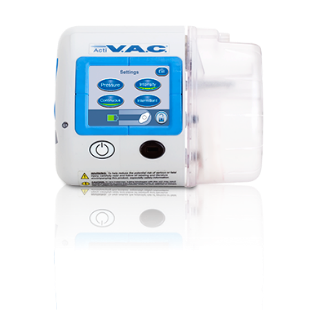ACTIVAC (KCI) | - Has rechargeable battery that lasts for 14-hours and that takes about 6-hours to charge. - When an alert sounds, you can press audio pause to silence the sound. The tone will come back if you have not fixed the problem within two minutes. - Indicated for wounds with low exudate (less than 300mL over 3 days). |

| **SELF-CONTAINED DEVICES** | Description |
| --- | --- |
| PICO 7 (Smith & Nephew)  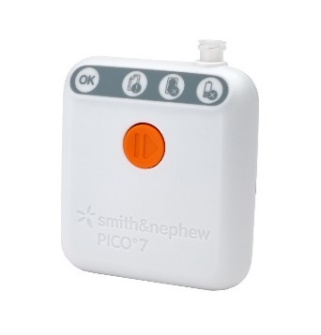 | - Disposable and canister free. - Powered by two AA lithium batteries. - Indicated for wounds with low exudate (less than 300ml of exudate a week). - Not usually applied on hands and feet. |
| 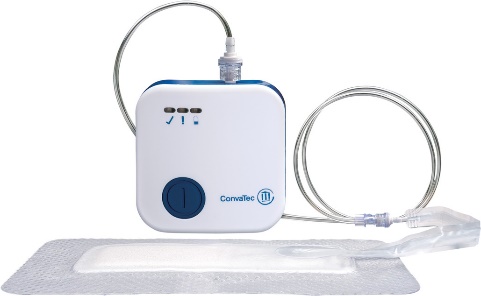AVELLE (Convatec) | - Has visual indicators to show the pump is functioning correctly. - There are no audible alarms. - It is powered by three AAA lithium batteries. |

1. **COMMENCEMENT OF NPWT**

**Obtaining/returning a NPWT device:**

- [AS PER LOCAL HHS WORKFLOW, MAKE SURE TO INCLUDE AFTER-HOURS]

**Prior to commencing the dressing ensure:**

- The patient has had appropriate first aid and pain relief has been administered
- Perform hand hygiene and ensure appropriate PPE is worn
- Prepare the wound as per hospital policy

**6.1 CANISTER DEVICES (e.g., RENASYS TOUCH/GO, KCI)**

**Equipment needed:**

- Pump with charger
- Cannister
- Soft Port/Port
- ‘Y’ connector if connecting two dressing to one device
- EasyDress sleeve if applying to complex area (e.g., hand or foot)
- Film
- Kerlix
- Primary dressing as per hospital policy (e.g., INSERT LOCAL HHS primary dressing)
- Scissors

| **Steps** | **Additional Information** |
| --- | --- |
| 1. **Apply dressing**  - Apply primary dressing - Secure with hypafix to reduce movement, if needed - Cover primary dressing with layers of kerlix gauze - Cover dressing with plastic film, ensuring all areas are fully covered - If using EasyDress sleeve, place sleeve over hand or foot. Seal the opening of the sleeve using the film provided. | There are no set amounts of layers to apply, however enough is needed to absorb any exudate.  Alternatively you can use tegaderm without the hypafix edging to cover the dressing.  Avoid stretching or pulling the transparent film to minimise tension or trauma to the peri-wound skin. |
| 1. **Apply port**  - Cut a hole in the centre of the dressing, approximately the size of a 20c piece - Remove backing of the port and apply over the hole created | Ensure you line the hole of the soft port up with the hole of the dressing.  Take time to think about where the port is going to make it easy for parents to dress their child while using the pump. |
| 1. **Connect**  - Connect the canister to the pump and click into place, connect port to the canister tubing |  |
| 1. **Turn machine on**  - Ensure the affected area is in the correct position - Turn on the pump and select settings - Once set, seal connections between soft port and cannister to prevent disconnection during treatment using clear film | This is most likely 80mmHg however please consult the burns team if unsure.  You should notice the air being sucked out of the dressings while the machine checks for leaks. |

**6.2 TROUBLESHOOTING**

Below are the most common warning signals that will alarm on the device. If the device is showing an alarm that is not on this list, please consult the troubleshooting guides and the end of the document under **Additional Resources**.

| **Warning Signal** | **What to do** |
| --- | --- |
| Leak/Low Vacuum | - Check the tubing and make sure they are properly locked - Check the edges of the machine to see if any film has lifted - There may also be a hole in the soft port - You often might hear a ‘hissing’ sound where the air is leaking from - Use the film to seal the edges of the dressing or around the soft port. - Once sealed, the machine should correct itself   **You can also use the SEAL CHECK Leak Detector:**   - Listen for a hiss and move your hands around the edge of the dressing to try and find the leak - The bar graphs will get shorter and the alarm tone with decrease when the leak is found - Reinforce edges of the dressing with the clear adhesive tape |
| Blockage/Full | - Check the tubing for kinks and lower the device and tubing below the wound site - The canister may be full or there may be condensation in the tubing which will require changing of the canister - if there is excessive exudate then the cannister could be full, the liquid in the cannister may have splashed over the filter registering a full cannister or the solidifier within the cannister has opened up and blocked the filter registering a full cannister - To correct this, a new cannister needs to be applied |
| Low Battery | - Plug the device into the external charging cord - Ensure that the cord is correctly placed into the side of the machine - The Battery indicator light should slowly flash green when charging and will be solid green when charging is complete |

**6.3 SELF-CONTAINED DEVICES (e.g. PICO 7, AVELLE)**

Equipment needed:

- Pump with batteries
- Dressing pack (an appropriate size that covers the burn)
- Film or sleeve
- Primary dressing as per hospital policy (e.g., INSERT LOCAL HHS primary dressing)

| **Steps** | **Additional Information** |
| --- | --- |
| 1. **Apply dressing**  - Apply primary dressing - Secure with hypafix to reduce movement, if needed - Remove the plastic backing from the PICO dressing and place centrally over the wound - Smooth the dressing around the wound to prevent creasing | Ensure the dressing is applied with tubing on the higher end of the wound as ooze from the wound pooling around the port can cause a blockage |
| 1. **Seal and connect**  - Seal around the edges of the dressing with the extra film strips supplied within the dressing pack - Connect the dressing to the tubing from the pump |  |
| 1. **Turn machine on**  - Ensure the affected area is in the correct position - Inset batteries into the device - Switch on by pressing the orange button on the pump, all three lights should flash at one. | You should notice the air being sucked out of the dressings while the machine checks for leaks. |

**6.4 TROUBLESHOOTING**

Below are the most common warning signals that will alarm on the device.

If the device is showing an alarm that is not on this list, please consult the troubleshooting guides and the end of the document in **Additional Resources.**

| **Symbol** | **What to do** |
| --- | --- |
| 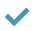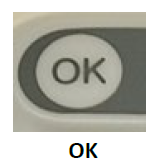 | - The green OK symbol will flash green continuously when everything is working correctly - No action required |
| 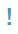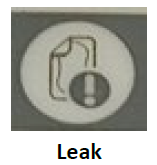 | - Make sure the dressing is properly stuck down and smooth out any creases - Reinforce edges of the dressing with the clear adhesive tape - The AVELLE pump will turn off after 30 seconds. Then press the blue button for 3 seconds to re-start the device and the green “✓” indicator light should start to flash again |
| 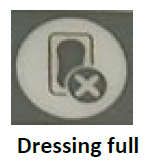 | - If the dressing is saturated, no pressure is being applied - An orange dressing full indicator will flash - You may need to change the dressing |
| 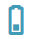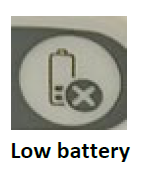 | - The batteries should last one week. - Low battery will only happen if there is a leak and the pumps motor is working extra hard - The batteries may need to be replaced and the leak sealed |

1. **REMOVAL OF DRESSING**

The NPWT dressing is to remain in situ for a minimum of 3 days and maximum of 7 days – unless clinical decision from treating team to cease earlier. If ceasing NPWT prior to the minimum timeframe, documentation is required as to clinical reasons for protocol deviation. The NPWT dressings should be reviewed by the clinical team for possible removal of dressing if there is excessive exudate on the dressing or in the canister (for canister devices) – or if there is more than 75% strikethrough on the dressing (for self-contained devices). NPWT is a one-off adjunctive treatment and does not need to be reapplied after 3 – 7 days of treatment under the INPREP Trial Pathway. When removing the NPWT dressing:

- Turn machine off (the pump and batteries may be placed in recycled waste as non-clinical waste if seven days old if using PICO devices)
- Disconnect tubing from the dressing – if using extension tubing
- Remove dressing and fixation strips gently (use remove wipes if necessary)
- Dispose of dressing into clinical waste (including tubing if day seven)
- Cleanse and assess wound, apply new primary dressings (if required) as per standard care (e.g., INSERT LOCAL HHS primary dressing)

1. **COMPLETION OF DRESSING**

On completion of the dressing, it is helpful to reinforce to families the following points and provide them with a Negative Pressure Wound Therapy fact sheet:

- Patients should not shower while connect to negative pressure wound therapy
- The pump should remain in an upright position when used
- Provide family with extra clear film and tape for dressing
- No sand, dirt, or water
- Avoid getting hot/sweaty
- To return to emergency if any signs or symptoms of infection including rash, diarrhoea, vomiting, temperature, cough/runny nose or not eating or drinking.
- If any concerns, please contact Burns/Surgical team

**Additional Resources**

**[*Remove NPWT devices not available within your local HHS*]**

RENASYS

- Smith and Nephew Wound: RENASYS Instructional Video: [How to apply RENASYS Soft Port NPWT to Wound on the Heel - YouTube](https://www.youtube.com/watch?v=qlPpXzGCNqo)
- RENASYS TOUCH Troubleshooting [(2) RENASYS TOUCH Troubleshooting - YouTube](https://www.youtube.com/watch?v=BzqFrcpv9_Y)
- Troubleshooting - RENASYS GO [NPWT Troubleshooting Website: Troubleshooting - RENASYS GO (npwtsupport.co.uk)](http://npwtsupport.co.uk/new/troubleshoot-rgo.php)

KCI

- National Wound Care: KCI Instructional Video: [NPWT Training - How to apply dressing for negative pressure wound therapy - YouTube](https://www.youtube.com/watch?v=-0eeoacEw-k)
- 3M Health Care: ActiV.A.C Therapy System Clinician Instructional Video: [3M™ ActiV.A.C.™ Therapy System Clinician Instructional Video - YouTube](https://www.youtube.com/watch?v=bQ3mPVM8RMs)
- ACTIV.A.C.™ Therapy System Alarm Troubleshooting <https://multimedia.3m.com/mws/media/2155847O/activ-a-c-therapy-system-alarm-troubleshooting-guide.pdf>

PICO

- PICO application and troubleshooting videos [Application videos | Possible with PICO](https://www.possiblewithpico.com/application-videos)

AVELLE

- A Guide on How To Use The Avelle™ Negative Pressure Wound Therapy System For Shallow Wounds [A Guide on How To Use The Avelle™ Negative Pressure Wound Therapy System For Shallow Wounds - YouTube](https://www.youtube.com/watch?v=rBvY_F9UNy0)
- CONVATEC – instructions for use and troubleshooting [SI2216F Avelle IFU-page1 (avelle-npwt.com)](https://avelle-npwt.com/media/q5dfrhsz/si2216f-avelle-ifu-page-1.pdf)

NPWT INSTRUCTIONAL VIDEO


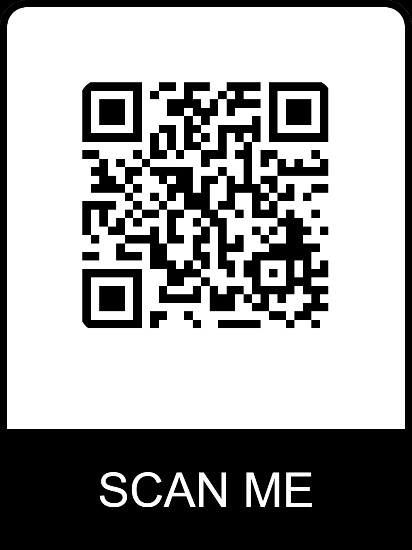


REFERENCES

1. Frear CC, Cuttle L, McPhail SM, Chatfield MD, Kimble RM, Griffin BR. Randomized clinical trial of negative pressure wound therapy as an adjunctive treatment for small‐area thermal burns in children. British journal of surgery. 2020;107(13):1741-50.
