# Supplementary material for: Implementation of negative pressure for acute pediatric burns (INPREP): A stepped-wedge cluster randomized controlled trial protocol
Source: PLoS One. 2024 Dec 10;19(12):e0315278. doi: 10.1371/journal.pone.0315278 (PMC11630585; doi:10.1371/journal.pone.0315278)
Supplement: S3 File — (DOCX) [file pone.0315278.s004.docx]

**Negative Pressure Wound Therapy – Parent Guardian Education Sheet**

Negative Pressure Wound Therapy (NPWT) uses a vacuum machine, sometimes called a vacuum pump, that is applied on top of regular burns dressings. The machine provides a gentle suction that helps burn wounds to heal faster. This treatment also helps:

-
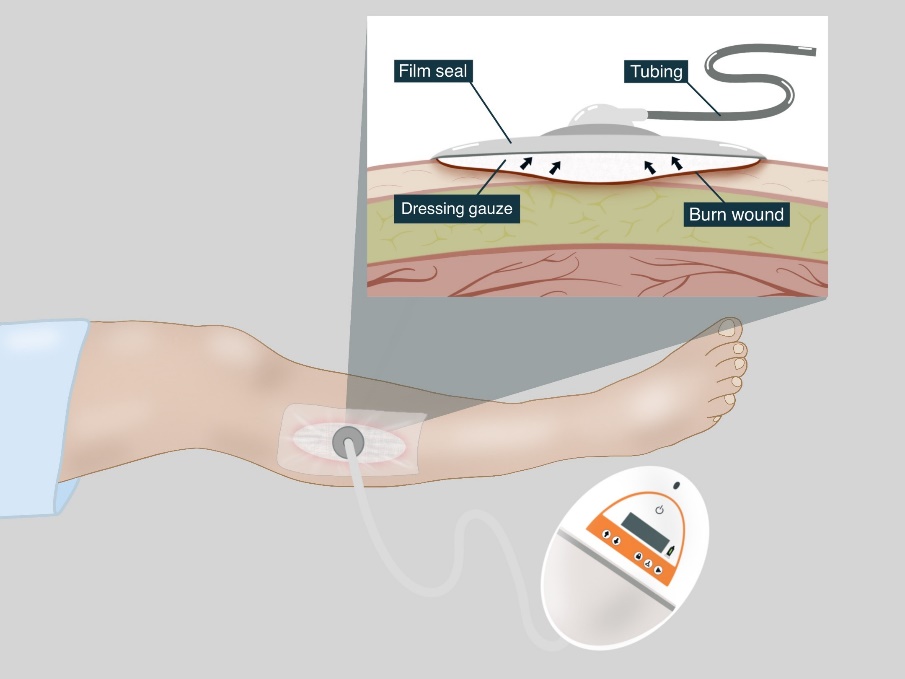
Remove excess fluid from the burn wound
- Promotes growth of new skin cells by improving blood flow to the wound area
- Pull the burn wound edges closer together

Your child has received NPWT because your doctor feels that their burns might benefit from this therapy.

**Please contact us immediately if:**

- There is a sudden increase or large amount of **blood or fluid** on the dressing or within the canister
- The dressing feels or appears **loose**
- Your child has a **fever**
- There is an increase in **pain**, **redness**, or **smell**
- The device keeps flashing as an **alarm** and/or **continually buzzing that you are unable to stop at home**

**In-Hours & After-Hours Contact Details**

7.30AM – 4PM Monday to Friday:

- Burns Unit Address [INSERT LOCAL ADDRESS]
- Burns Unit Number [INSERT LOCAL NUMBER]
- Burns Unit Email [INSERT LOCAL EMAIL]

After-Hours & Weekends:

- Call [INSERT LOCAL SWITCH NUMBER] and ask for the [Surgical/Plastics] Registrar or Burns Registrar on call

**Important things to note:**

- The unit should always remain upright
- The dressing must stay on until your child returns to the hospital for a wound review
- The pump's tubing could be considered a choking hazard and should be kept free from the face and neck area
- Do not disconnect the tubing
- Never turn the machine off without seeking medical advice from the hospital
- Always bring the charger and power cords to your appointments

**Fall Prevention Tips**

- Place the electrical cord extra tubing so that it is not a tripping hazard. When the machine is off or running on the battery unplug the cord and put it away
- Be aware of doorknobs or other objects that could catch on the tubing
- Use extra caution when your child is getting in and out of bed and assist them when needed

**Frequently Asked Questions**

1. ***How long it will the negative pressure wound therapy be applied for?***

We are using negative pressure wound therapy as a one-off (i.e., applied only once) adjunctive treatment. This means that negative pressure wound therapy is applied on top of our normal standard burn dressings. Negative pressure wound therapy will be applied once as part of your child’s acute burn care – and will be left on for 3 - 7 days.

1. ***What does negative pressure wound therapy look like when it is working?***

The negative pressure wound therapy dressing will shrink when it is working. The film will look wrinkled but feel firm to touch.

1. ***What does negative pressure wound therapy feel like?***

Most patients who use negative pressure wound therapy say it is not painful. It may cause a mild “pulling” feeling that goes away after a few minutes. If your child is experiencing on-going pain, speak to your nurse or doctor. They may look at changing the settings or prescribing pain relief.

1. ***Can my child take a shower or bath with the negative pressure wound therapy?***

Please do not get the negative pressure pump wet. This includes taking a shower or bath with the device. Sponge bath your child while they are wearing the pump.

1. ***Should the device make noise?***

When the device is on, you may hear a small amount of noise. This is because the device has moving parts inside. Placing the device below the level of the wound may help the device work more efficiently and reduce noise. If the noise becomes louder, or an alarm sounds, you may have a leak or a poor seal.

1. ***Can my child move around while receiving negative pressure wound therapy?***

The device is lightweight and designed to be portable to allow your child to move around. However, your child’s ability to move around depends on their wound location and other instructions from their medical team. If you are unsure, please ask their nurse or doctor.

**Alarms & Troubleshooting**

The negative pressure wound therapy machine will sound or flash an alarm if there is an issue. There are three main reasons the NPWT machine will alarm:

1. **Leak** – this means the suction is low because there is a leak in the seal of the dressing
2. **Dressing or canister is full –** the dressing over the burn and/or canister is saturated, and no suction is being allied
3. **Low battery power –** battery is low.

If the alarm continues after following the troubleshooting steps, **please contact the hospital for advice.** There may be a potential issue with the dressing, NPWT device, or canister.

[REMOVE DEVICES NOT AVAILABLE AT YOUR HHS]


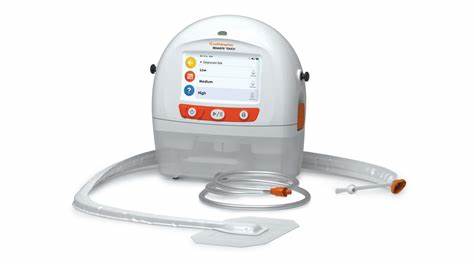
RENASYS GO/TOUCH Devices

-
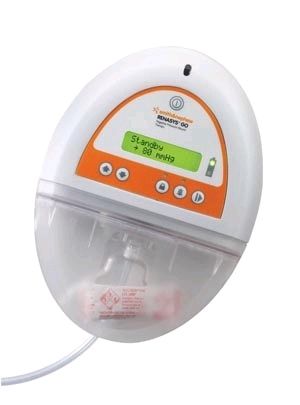
The device-battery will only last for approximately 20-hours. It is recommended to always charge overnight, advised to plug in to top up battery whenever possible to avoid flat battery when mobile
- The device has both audible and visual alarms
- If the absorbent pillow inside the canister ruptures, this is not a concern

**Warning signals that may appear on the device:**

| **Warning Signal** | **What to do** |
| --- | --- |
| Leak/Low Vacuum | - Listen for the “hiss” sound - this indicates a leak. - If you find the source of the hiss, you will need to reinforce the dressing with clear adhesive tape (this will be provided to you). - If you can’t hear a hiss, you will need to tape around all edges of the dressing. - Also make sure the canister is connected to the device correctly. |
| Blockage/Full | - The canister may be full or there may be condensation in the tubing which will require changing of the canister. - This can only be done by the hospital. - To prevent this, keep out of steamy bathrooms. - Also check there are no kinks in the tubing. |
| Low Battery | - Plug the device into the external charging cord. - Make sure the other end of the cord is plugged into you wall outlet and the power is on. - The Battery indicator light should slowly flash green when charging and will be solid green when charging is complete. |

PICO 7 Device

-
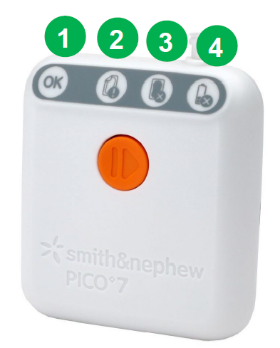
It is powered by two lithium AA batteries
- The PICO contains a magnet. Please advise your doctor or nurses if any person that will be close to the pump has an implantable medical device
- The PICO has no audible alarms, but the pump may be heard running occasionally as it maintains the negative pressure. This is normal.

**Warning signals that may appear on the device:**

| **Number** | **Symbol** | **What to do** |
| --- | --- | --- |
| **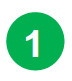** | 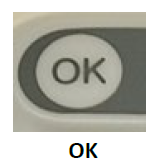 | - The green OK symbol on the front of the PICO 7 will flash green continuously when everything is working correctly - No action required |
| 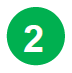 | 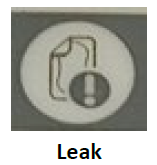 | - If an air leak has been detected, an orange leak indicator will flash - Reinforce edges of the dressing with the clear adhesive tape provided on discharge |
| 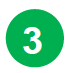 | 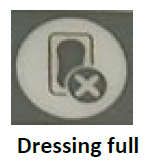 | - If the dressing is saturated, no pressure is being applied - An orange dressing full indicator will flash - Please contact the hospital to arrange to have the dressing checked |
| 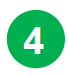 | 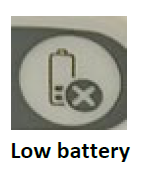 | - On the front of the pump, the green OK indicator and orange battery low indicator will flash together when the batteries need changing - This will only happen if there is a leak and the pumps motor is working extra hard - Please contact the hospital to check for leaks and replace the battery |


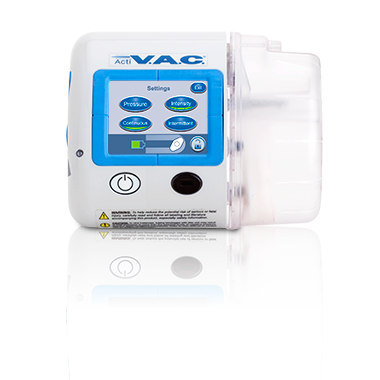
KCI/ACTIVAC Device

- The unit contains a rechargeable battery that lasts for 14-hours and that takes about 6-hours to charge
- It is recommended to always charge overnight, advised to plug in to top up battery whenever possible to avoid flat battery when mobile
- When an alert sounds, you can press audio pause to silence the sound. The tone will come back if you have not fixed the problem within two minutes

**Warning signals that may appear on the device:**

| **Warning Signal** | **What to do** |
| --- | --- |
| Leak | - Check the tubing and make sure they are properly locked. - **You can also use the SEAL CHECK Leak Detector:** - Listen for a hiss and move your hands around the edge of the dressing to try and find the leak. - The bar graphs will get shorter and the alarm tone with decrease when the leak is found. - Reinforce edges of the dressing with the clear adhesive tape provided on discharge. - If the leak is not fixed, therapy may stop. |
| Blockage alert/Low pressure alert | - Check the tubing for kinks and lower the device and tubing below the wound site. |
| Low Battery | - The battery level is shown on the bottom of the touch screen. - The battery light will glow green when fully charged, amber when the battery is low and red when the battery is critically low. - A battery low alert will appear on the screen two hours before the battery runs out. - To charge, plug the device into the external charging cord. Make sure the other end of the cord is plugged into you wall outlet and the power is on. |

**
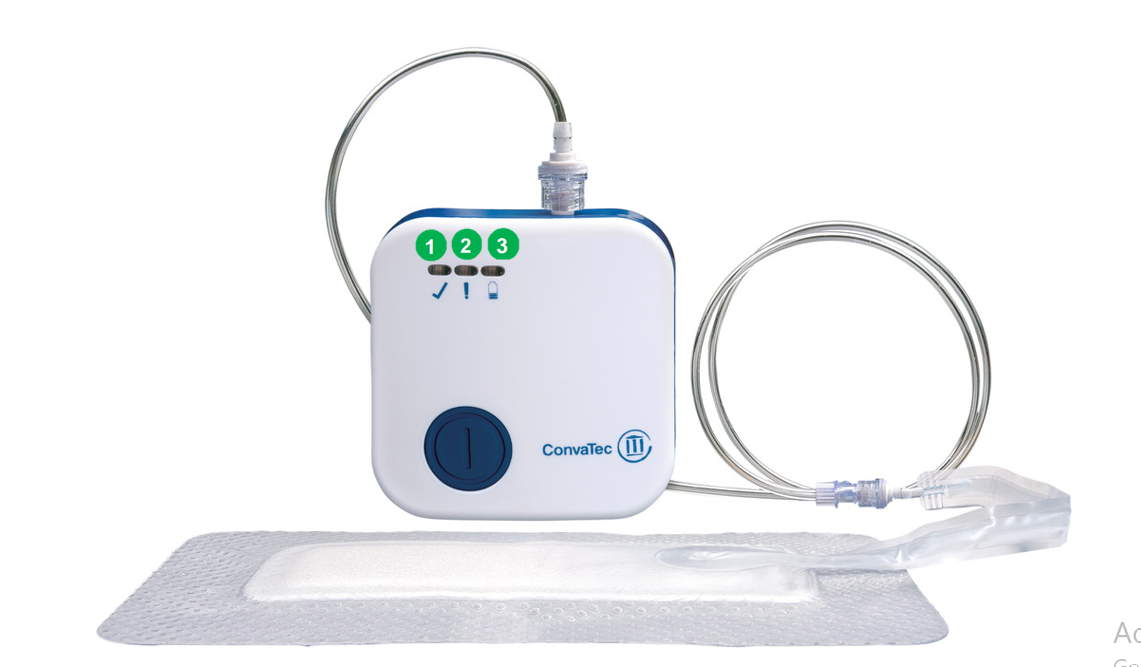
**AVELLE DEVICE

- The Avelle device is fitted with visual indicators to show the pump is functioning correctly – there are no audible alarms
- It is powered by three AAA lithium batteries

**Warning signals that may appear on the device:**

| **Number** | **Symbol** | **What to do** |
| --- | --- | --- |
| **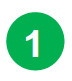** | 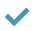  OK | - The green light above this symbol on the Avelle NPWT device will flash green continuously when everything is working correctly - No action required |
| 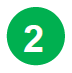 | 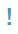  AIR LEAK | - If an air leak has been detected, the yellow light above this symbol will flash - The pump will turn off after 30 seconds - Make sure the dressing is properly stuck down and smooth out any creases - Reinforce edges of the dressing with the clear adhesive tape provided on discharge - Then press the blue button for 3 seconds to re-start the device and the green “✓” indicator light should start to flash again |
| 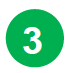 | 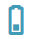  LOW BATTERY | - The yellow light above this symbol will flash if the battery is low and needs changing. - This will only happen if there is a leak and the pumps motor is working extra hard - Please contact the hospital to check for leaks and replace the battery |
