# Supplementary material for: Implementation of negative pressure for acute pediatric burns (INPREP): A stepped-wedge cluster randomized controlled trial protocol
Source: PLoS One. 2024 Dec 10;19(12):e0315278. doi: 10.1371/journal.pone.0315278 (PMC11630585; doi:10.1371/journal.pone.0315278)
Supplement: S4 File — (DOCX) [file pone.0315278.s005.docx]

### Supplementary File 4. Wound Fluid Collection, Processing, and Storage

*Methods taken from: SOP-Wound-Fluid-v3-20200212*

**Purpose**

This SOP describes the methodology for the collection and processing of burn wound fluid (WF) samples. Wound fluid samples are opportunistically collected from burn patients who present to the burn center or emergency department with a burn injury. As part of routine clinical wound cleaning procedures, blisters are de-roofed, and blister fluid is removed and discarded. For this project, instead of being discarded, the blister fluid will be collected, processed and stored so that it can be analyzed and used for burn wound healing studies. Additionally, if patients present with an exudating wound, this fluid will be collected instead of discarded. And if patients present with a negative pressure device attached to their wound, and there is fluid in the suction tube, this will also be collected. Samples are ideally received in volumes >100uL, although occasionally less is collected, as sample volumes as small as 50µL can still be analyzed using mass spectrometry.

**Aim**

To process WF specimens and freeze in smaller aliquots to maintain protein quality and prevent repeated freeze thawing of samples.

**Reagents and Consumables**

- Specimen pot 70mL, Sarstedt Cat#75.9922.745 (Polypropylene)
- Whatman Filter paper, sterilized
- Eppendorf Protein Lo-bind tubes Cat#30108094
- Filtered pipette tips
- Pipettes – P200 and P1000
- Microcentrifuge, for 1.5mL to 2.0mL tubes
- Site-specific participant code book or REDCap database
- De-identified wound fluid sample logbook or REDCap database
- Brady label maker Cat#BMP51

Standard Personal Protective Equipment (PPE) must be worn at all times when collecting and processing biological specimens, including gloves and safety glasses during collection and gloves, safety glasses and a laboratory gown when processing the samples in the laboratory. Any staff member or student who is involved in the processing of biological samples must be immunized against Hepatitis B.

**Procedure**

1. During routine wound cleaning procedures, the clinical/nursing team will lance patient blisters and remove any exudating wound fluid. If there is a large volume of fluid (e.g., several mLs), the fluid can initially be collected in a larger container, such as a specimen pot temporarily.
2. If the samples are smaller volume, they can be collected directly into the Eppendorf Protein Lo-bind tubes.
3. If there are multiple blisters on the same patient, these should be collected in separate tubes/pots.
4. If the patient has a negative pressure device with fluid in the tubing, the tube can be clamped at either end, cut off the device and the fluid from the tube collected in a specimen pot.
5. If there is only a small amount of fluid, this can be collected with filter paper. The filter paper is placed at the wound edge to absorb the fluid, and then placed into sterile 2mL Lo-Bind tubes.
6. If a sample is collected in a specimen pot, please transfer to a Lo-bind tube ASAP to prevent protein loss.
7. Label the tube/pot with patient UR sticker, the anatomical location of the blister or wound site (e.g., right palm, left thigh) and collection date. **Store in a fridge at 4°C if not processed immediately, and process within 24 hours.**
8. If the sample is in a specimen pot, transfer it into Eppendorf Protein Lo-bind tube/s ASAP. If you transfer it into multiple tubes (i.e., the volume is greater than 2.0 mL), transfer equal amounts into each tube, so that they will balance in the centrifuge. Label the tube with the participant unique identifier code from your site-specific participant code book or from the REDCap database.

e.g., 49_ 23_ WFa_20190408

(Brisbane Code_ Participant #23_ Wound Fluid__Anatomical Area abcde_Date yyyymmdd)

1. If the sample is in filter paper, recover the wound fluid by eluting the sample in 100µL of Phosphate Buffered Saline or water at 4◦C for 1 hour (45).
2. Centrifuge the sample in a balanced benchtop microfuge for 3 minutes at 855 x g at ambient temperature to pellet cells and cellular debris.
3. Record the collection date, anatomical location, total sample volume and any comments on the fluid contents (Blood? Color? Consistency?) in the WF sample logbook or in the REDCap database.
4. Without disturbing the pellet, transfer the supernatant to fresh Lo-bind tubes. The volume in each tube should be minimum 50µL, to a maximum of 1000µL. Ensure there are at least 2 aliquots (2 tubes) of each sample. Write the total number of aliquots and aliquot volumes in the WF sample logbook or REDCap database.
5. Label each aliquot with sample number and date, using the Brady label maker.
6. Store aliquots in a -80°C freezer.
