# Supplementary material for: Implementation of negative pressure for acute pediatric burns (INPREP): A stepped-wedge cluster randomized controlled trial protocol
Source: PLoS One. 2024 Dec 10;19(12):e0315278. doi: 10.1371/journal.pone.0315278 (PMC11630585; doi:10.1371/journal.pone.0315278)
Supplement: S5 File — (DOCX) [file pone.0315278.s006.docx]

### Supplementary File 5. Blood Collection, Processing, and Storage

*Method taken from: SOP-Blood-Processing-5-2020200421*

**Purpose**

This SOP describes the methodology for the collection and processing of blood samples. Blood samples are opportunistically collected from burn patients who are undergoing a GA for routine burn injury treatments (e.g., dressing change, debridement, or grafting). The volume of blood that can be collected from a child depends on their age/size and total blood volume. According to the WHO guidelines for the safe limits of blood volume collection from children, 1 – 5% of total blood volume can be collected within a 24-hour period, with a maximum of 3mL per kg for sick children. To obtain enough cells to process, we will collect a minimum of 1mL blood volume, and ideally collect around 8-10mL.

Blood will initially be collected in a heparinized tube. A small amount of whole blood will be removed and prepared for transcriptomic (gene expression), epigenetic and immune phenotypic assays. The remaining sample will be processed through several centrifugation steps, and Peripheral Blood Mononuclear Cells (PBMCs) and plasma will be isolated. The plasma, whole blood isolates and cells will be aliquoted into tubes. Plasma and whole blood samples will be stored at -80◦C and cells will be stored in liquid Nitrogen in a research facility or hospital laboratory at each site. No more than 5% of the whole blood volume will be removed in a single occasion. In cases where pediatric patients do not require blood sampling, canulation, or a GA as part of their routine standard care, blood collection will not be performed. All patients will be offered a numbing cream to help reduce pain during blood sampling if there are concerns regarding discomfort.

**Aim**

To collect and process blood specimens to obtain Peripheral Blood Mononuclear Cells (PBMC) and plasma. These samples will then be frozen for later analysis.

**Reagents and Consumables**

- Vacutainers containing lithium heparin (e.g., Becton Dickinson green 10mL Cat #367874)
- Eppendorf Protein Lo-bind tubes Cat#30108094
- Lymphoprep or Ficoll-paque Plus – brought to room temperature
- RPMI containing 20% Heat-inactivated fetal calf serum FCS/FBS – brought to room temperature
- 15mL and 50mL polypropylene tubes
- Sterile, filtered pipette tips
- Pipettes – P200 and P1000
- Electronic pipette aid and serological pipettes (2mL – 25mL)
- Vacuum suction and glass pipettes
- DMSO
- Cryovials, 2ml Corning CLS430659, these cryovials are for LN2 Vapor storage.
- CoolCell/MrFrosty
- Site-specific participant code book or REDCap database
- De-identified blood sample logbook or REDCAP database
- Brady label maker Cat#BMP51

Standard Personal Protective Equipment (PPE) must be worn at all times when collecting and processing biological specimens, including gloves and safety glasses during collection and gloves, safety glasses and a laboratory gown when processing the samples in the laboratory. Any staff member or student who is involved in the processing of biological samples must be immunized against Hepatitis B.

**Procedure – Plasma and PBMC**

1. Collect blood into vacutainers containing lithium heparin (the BD vacutainers we use specify “17 international units of heparin/mL of blood”). Aim to collect between 8-10mL of blood. Label tube with participant unique identifier code from your site-specific participant code book or REDCap database, and collection date and time. **Process immediately if possible. Store at room temperature for up to an hour, for longer storage than an hour place in the fridge, and process within 24 hours.**
2. Record the collection and processing date and time in your site-specific participant code book or REDCap database. Centrifuge blood at 400 x g for 10min, brake ON. (400G=1340RPM, where radius=20cm)
3. Aliquot the plasma supernatant into 3-4 x Eppendorf Lo-bind tubes and label these with the unique patient code from your site-specific patient code book or REDCap database, using the Brady label maker.

e.g., 49_23_ P_20190408 (Brisbane Code_Participant #23_Plasma_Date yyyymmdd)

1. Record the collection date and total plasma volume in the blood sample logbook or REDCap database. Store plasma aliquots in a -80 freezer.

Vacutainer Plasma & Blood Fractions:


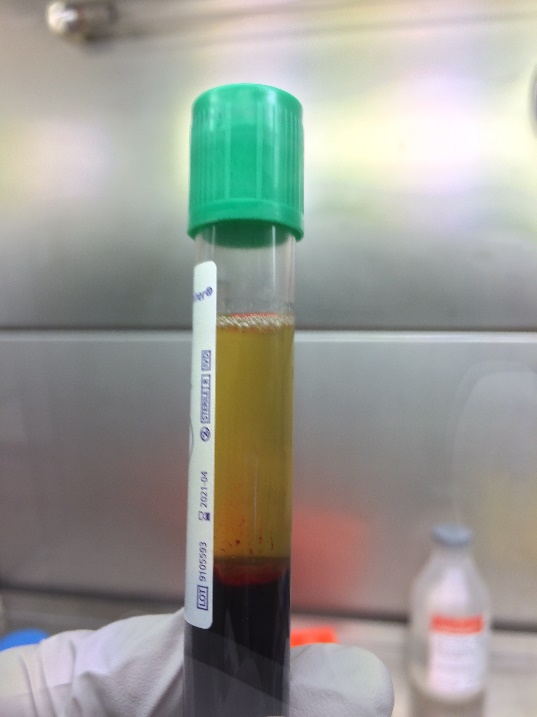


Plasma fraction for collection. Do not disturb interphase with RBCs, leave some plasma behind in tube

1. Dilute the remaining blood cells up to 4x in room temperature RPMI+20%FCS. It will probably be necessary to make a 2x dilution first, transfer to a 10 or 15mL tube, then dilute 2x again. * Dilute the remaining cells by first replacing the volume of plasma you removed, i.e. If you removed 4ml of plasma – place 4mls of RPMI & FBS into the remaining blood sample. Secondly, dilute the whole sample 1:1 with RPMI&FBS.
2. Prepare 2 x 15mL tubes by pipetting the correct volume of room temperature Ficoll-paque Plus (or Lymphoprep). See below for volumes (note: use regular 15ml tubes in place of Sepmate tubes):

**Table 1.** Sample and Density Gradient Medium Volumes

| SEPMATE^TM^ TUBE | INITIAL SAMPLE (mL) | DENSITY GRADIENT MEDIUM (mL) |
| --- | --- | --- |
| 15 | 0.5 – 4.0 | 4.5 |
| 15 | >4 – 5 | 3.5 |
| 50 | 4 – 17 | 15 |

1. Split the diluted blood equally between the two tubes, carefully pipetting down the sides of the tubes.

Blood & Lymphoprep Fractions Pre-centrifugation


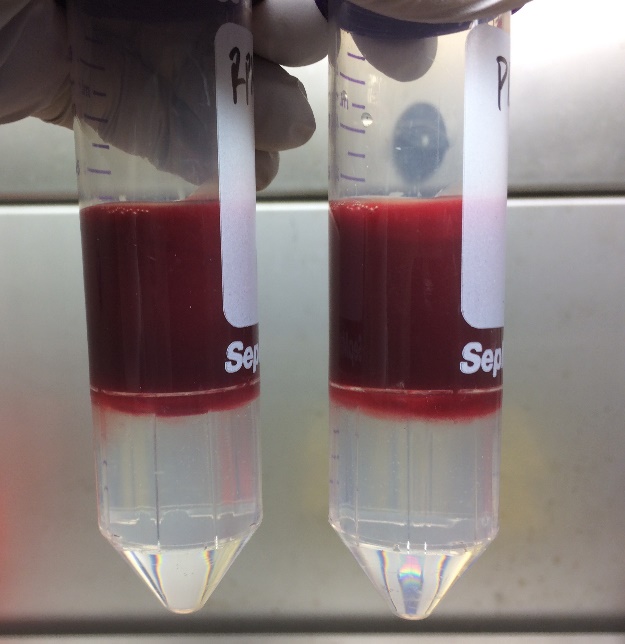


1. Centrifuge the sample at 400 x g for 30min at room temperature with the **brake OFF**. (400G=1340RPM, where radius=20cm)

Tubes post spin should look similar to these:


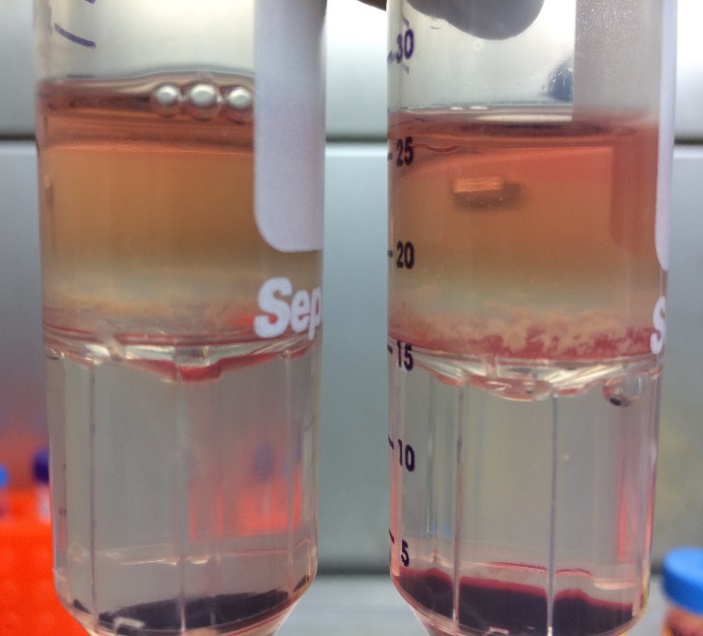

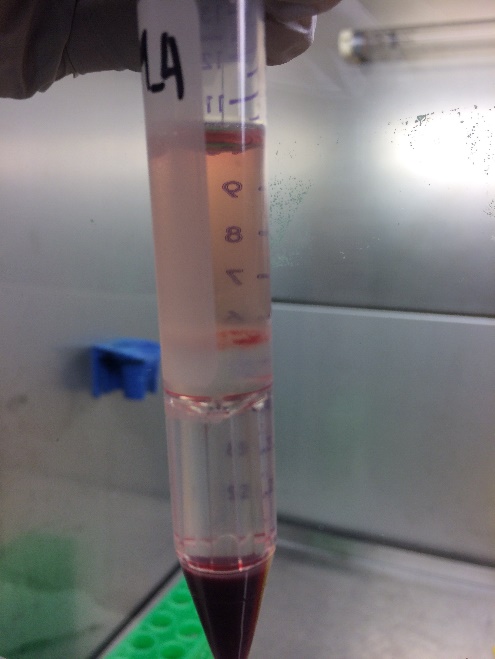


Clear PBMC fraction to collect

After centrifugation

1. For isolation of the buffy coat layer (containing PBMCs), carefully insert your pipette down the side of the tube and remove out the buffy coat layer. Resuspend into 2 x 15mL tubes.
2. Make each tube up to 15mL with RPMI + 20% FCS. Centrifuge at 500 x g for 10min, brake on. (500G=1500RPM, where radius=20cm)

PBMCs should be a pellet like this:


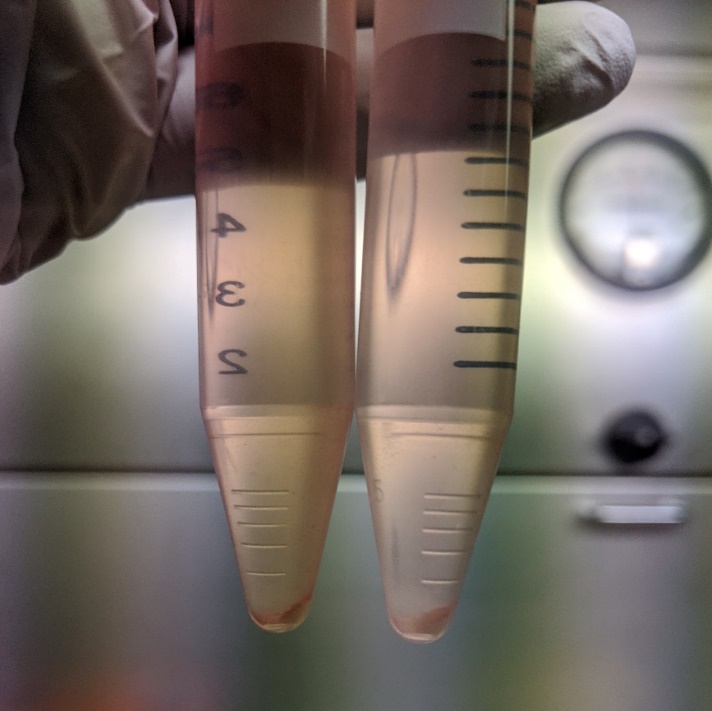


1. Discard the supernatants. Resuspend the pellet from one tube in 1mL of RPMI + 20% FCS and transfer to the other tube. Resuspend the cells in 15mL of RPMI + 20% FCS and centrifuge at 120 x g for 10min, **brake OFF.** This will deplete the platelets from the samples.
2. Discard the supernatant and resuspend the cells in 1mL RPMI + 20% FCS. Take an aliquot for counting.
3. The method used to count the cells is not important as long as it is accurate and allows for live/dead discrimination. We will count by taking a 1:10 dilution of cells, further diluting them 1:2 in 0.4% trypan blue, then counting manually using a hemocytometer.
4. Cells will be stored at approximately 2 x 10^6^ cells/vial. Using the label maker, label enough cryovials with the participant identifier code and place them on ice.

e.g., 49_ 23_ C_20190408 (Brisbane Code_Participant #23_Cells_Date yyyymmdd)

1. Dilute the cells with RPMI + 20% FCS to a concentration of approximately 2 x 10^6^ cells/mL per vial.
2. Place the cells on ice and add an equal volume of pre-chilled RPMI + 15% DMSO; take at least 1min to add the first mL of 15% DMSO drop-by-drop, and slowly add the rest.
3. Transfer aliquots to the labelled cryovials.
4. Move cryovials into a CoolCell/MrFrosty (at room temp) and store in a -80C freezer, at least overnight.
5. Transfer cryovials to liquid nitrogen storage. Write details of the number of cryovials in the blood sample logbook or REDCap Database.
