# Supplementary material for: Implementation of negative pressure for acute pediatric burns (INPREP): A stepped-wedge cluster randomized controlled trial protocol
Source: PLoS One. 2024 Dec 10;19(12):e0315278. doi: 10.1371/journal.pone.0315278 (PMC11630585; doi:10.1371/journal.pone.0315278)
Supplement: S6 File — (DOCX) [file pone.0315278.s007.docx]

### Supplementary File 6. Hair Collection, Processing and Storage

*Method based on Treya Long Protocol 2020*

**Collecting Hair Samples**

1. Separate the hair at the back of the head with a hair clip
2. Collect a hair strand of about 3-5mm and comb
3. Tie up the hair strand with a small hair band/tie or string close to the scalp
4. Cut the hair as close as possible to the scalp
5. Place the hair into the 2ml screwcap cryogenic tube

**Record Keeping**

Record the following in lab book:

1. Patient number
2. Date of sample processing
3. Time of sample processing
4. Name of scientist processing

**Hair Storage**

Tube should be labelled as follows:

- Patient Number_Sample Date (ddmmyyyy)_H

For example: 1_26032020_H)

- Hair should be stored directly at -80°C
