# Supplementary material for: Implementation of negative pressure for acute pediatric burns (INPREP): A stepped-wedge cluster randomized controlled trial protocol
Source: PLoS One. 2024 Dec 10;19(12):e0315278. doi: 10.1371/journal.pone.0315278 (PMC11630585; doi:10.1371/journal.pone.0315278)
Supplement: S7 File — (DOCX) [file pone.0315278.s008.docx]

### Supplementary File 7. Urine Collection, Processing and Storage

**Purpose**

This SOP describes the methodology for the collection and processing of urine samples. Urine samples are opportunistically collected from burn patients who present to the burn center or emergency department with a burn injury. If participants are scheduled to receive a urinary catheter as part of routine standard treatment for their burns, some urine that will be retained for analysis. For participants who do not require a catheter, the child and parent/caregiver will be asked if they consent to providing a urine sample and will be given a urine sample pot container for collection. A minimum volume of 60mL is required for analysis.

**Aim**

To process urine specimens and freeze in smaller aliquots to maintain protein quality and prevent repeated free-thawing of samples.

**Reagents and Consumables**

- Sterile Specimen pot 250mL, Sarstedt Cat#75.9922.745 (Polypropylene)
- Eppendorf Protein Lo-bind tubes Cat#30108094
- Filtered pipette tips
- Pipettes – P200 and P1000
- Microcentrifuge, for 1.5mL to 2.0mL tubes and 50mL tubes
- Site-specific participant code book or REDCap database
- De-identified urine sample logbook or REDCap database
- Brady label maker Cat#BMP51

Standard Personal Protective Equipment (PPE) must be worn at all times when collecting and processing biological specimens, including gloves and safety glasses during collection and gloves, safety glasses and a laboratory gown when processing the samples in the laboratory. Any staff member or student who is involved in the processing of biological samples must be immunized against Hepatitis B.

**Procedure**

1. A mid-stream urine sample (if possible) will initially be collected in a sterile specimen pot.
2. Transfer the sample into multiple 50mL Falcon tubes.
3. Centrifuge the tubes at 1,500 × g for 10 minutes. Collect the supernatant and pass through a sterile 0.2 μm syringe filter to remove any remaining bacterial contamination and high molecular weight protein aggregates.
4. Transfer 1mL aliquots of the retentate of each sample into Lo-bind tubes. Label each aliquot with sample number and date, using the Brady label maker.

For example: 49_ 23_ U_20190408 (Brisbane Code_ Participant #23_ Urine_Date yyyymmdd)

1. Store aliquots in a -80°C freezer.
2. Write the total number of urine aliquots and aliquot volumes in the sample logbook or REDCap database.
